# Optimal Multi-Drug Therapies for Antimicrobial Resistance with Horizontal Transfer

**DOI:** 10.1007/s10957-026-03025-y

**Published:** 2026-05-22

**Authors:** Francesca Calà Campana, Gian Paolo Incremona, Franco Blanchini, Patrizio Colaneri, Giulia Giordano

**Affiliations:** 1 Dipartimento di Ingegneria Industriale, Università degli Studi di Trento, Trento, Italy; 2 Dipartimento di Elettronica, Informazione e Bioingegneria, Politecnico di Milano, Milano, Italy; 3 Dipartimento di Scienze Matematiche, Informatiche e Fisiche, Università degli Studi di Udine, Udine, Italy; 4 Dipartimento di Elettronica, Informazione e Bioingegneria, Politecnico di Milano and IEIIT, CNR, Milano, Italy

**Keywords:** Antimicrobial resistance, Biological systems, Model predictive control, Optimal control, 49J15, 49N90, 92C50, 92D25, 93C10, 93C15

## Abstract

The emergence of antimicrobial resistance (AMR) is a global health threat that can be mitigated through the careful planning of multi-drug therapies. We analyse a model that captures the interplay between the host immune system and susceptible and resistant bacterial populations, accounting for the use of antibiotics and for treatment-induced mutations and horizontal transfer phenomena that favour the growth of resistant populations. We extend the model to consider multiple antibiotics and all the ensuing possible combinations of resistance. We formulate an optimal control problem aimed at minimising the overall bacterial population size as well as the use of antibiotics, and we rigorously prove that an optimal solution exists, which can be thus computed numerically. For the considered optimal control problem, we analyse necessary conditions for optimality based on the Pontryagin minimum principle, as well as singular controls. We also discuss an alternative approach for treatment design through a model predictive control (MPC) scheme. Our computational experiments, which illustrate both the direct solution to the optimal control problem and the MPC approach, with realistic parameters from the biological literature and initial conditions chosen according to several different scenarios, validate our theoretical results and demonstrate the flexibility of the framework, which adapts the therapy to the presence and numerosity of resistant populations, so that the optimal treatment strongly depends on both the cost functional weights and the initial conditions for the system dynamics.

## Introduction and Motivation

Microorganisms such as bacteria, fungi, and parasites evolve and can develop mechanisms to become insensitive to the effects of antimicrobial agents. The resulting *antimicrobial resistance* (AMR) poses a significant threat to global health, as it makes standard treatments ineffective, leading to persistent infections, increased mortality, and higher healthcare costs; moreover, AMR reduces the effectiveness of essential medical interventions, such as surgery and cancer therapy, which rely on antimicrobial drugs to prevent infections. While susceptible bacteria are affected by antibiotics, resistant bacteria are shielded by protective mechanisms, such as preventing antibiotics from reaching their target, or modifying the antibiotic target [31]. The use of antibiotics is a fundamental selective pressure driving the emergence of resistance; in particular, the overuse and misuse of antibiotics, combined with the natural evolutionary mechanisms of bacteria, have accelerated the development of resistance, leading to a growing number of untreatable infections. With the rapid rise of resistant strains and the limited development of new antimicrobial agents, AMR has become a critical public health challenge that demands urgent and coordinated action: resistant bacteria are among the leading causes of death worldwide and are projected to cause 10 million deaths each year by 2050 [48, 62].

Mathematical modelling has emerged as an essential tool to understand and address the complexity of the AMR phenomenon [6, 9, 33, 37, 59]. Dynamic models can offer insight into the interplay among the key mechanisms underlying the emergence of resistance, predict its evolution, and evaluate the impact of various interventions and therapies before they are implemented. By integrating data [9] on antimicrobial usage, transmission dynamics, and population structure, mathematical models provide valuable understanding of the spreading of resistance. Furthermore, they serve as powerful tools for optimising treatment, informing public health policies, and guiding resource allocation efforts, both in the clinical practice and in general settings.

While some models focus on the transmission of resistance among hosts, particularly in hospital wards (see, e.g., [19, 49, 50, 66]), and thus enable the search for optimal treatment protocols at the population level [12], another body of literature addresses the emergence of resistance in bacterial populations within the host. A pioneering model for bacterial growth and resistance development in the host, in the presence of multiple antibiotics, can be found in [21], which also proposes optimal strategies to minimise the overall bacterial population size. Progressively more complex models have been developed to capture the onset and evolution of resistance in bacterial population, accounting for bacterial growth, mutation, and selection under antibiotic pressure. Major resistance mechanisms can be classified into intrinsic (the innate ability of some bacteria to diminish the antibiotic efficacy) and *acquired* either through random mutations or through *horizontal transfer* of resistance genes. Resistance genes can be acquired (i) directly from other bacteria via conjugation, or (ii) through bacteriophage-mediated transduction, or (iii) by incorporating DNA pieces from the environment via transformation; of the three mechanisms for horizontal gene transfer, conjugation is deemed to be the prevalent for pathogenic bacteria in humans [47, 58]. The importance of horizontal gene transfer in modelling AMR has been highlighted by several studies [7, 16, 22, 27, 63, 64, 66]; see also the survey [37]. The terms expressing horizontal gene transfer in mathematical models build upon earlier work modelling, more in general, plasmid transfer [40, 51, 56]. Other models of AMR in the host account for the important role of the immune system [3, 16, 17, 27], which suppresses bacterial populations regardless of their resistance properties. The effects of both the immune system and horizontal transfer phenomena have been rarely combined in modelling work: a notable exception is the model proposed in [16] and then reconsidered in [27]; however, such a model has not been leveraged to systematically suggest optimal antibiotic therapies.

Optimal control of compartmental models [10] is a precious mathematical formalism that can be used to optimise drug administration, maximising treatment efficacy while minimising the spread of resistance. Optimal control approaches to contrast and prevent AMR have gained growing interest lately and there has been an increasing focus on optimal AMR control, based on mathematical models with different features; see the recent works [2, 3, 22, 30, 32, 55, 67].

Here, we consider an AMR model that incorporates both horizontal gene transfer and the effect of the immune system, and we formulate and analyse an optimal control problem to design a drug administration strategy that clears the infection while preventing the spread of antimicrobial resistance. In particular, in Section 2, we introduce the considered single-antibiotic AMR model, which captures bacterial growth, mutations and horizontal transfer, along with the immune system response and the effect of antibiotic therapy with a single drug, to which bacteria can be either susceptible or resistant. Under biologically-grounded assumptions, we provide a thorough analysis of the model: we assess the system equilibria, their stability properties and their domain of attraction, and the qualitative behaviour of the system trajectories originating from different initial conditions, both in the absence of drug (Theorem 2.1) and in the presence of a constant drug concentration (Theorem 2.2); in particular, we identify a *safe set* from which all trajectories converge to the disease-free equilibrium *even for zero drug concentration*, and we pose the problem of finding a therapy that drives the system trajectories to such a safe set (Problem 2.1). Section 2.1 discusses the effect of including a logistic growth term. Section 3 extends the proposed model to the multiple-antibiotic case. In Section 3.1, we consider the case of two antibiotics, when bacteria can be susceptible to both antibiotics, or to just one of the two, or to none of the two, and we show that, although the model complexity increases, the main qualitative results achieved for the single-antibiotic case and the existence of a safe set are preserved. Then, in Section 3.2, we discuss the extension to an arbitrary number of antibiotics, and we showcase in particular the case of three antibiotics. In Section 4, we formulate and analyse an optimal control problem aimed at minimising a metric of interest that accounts for both the size of the bacterial population (which should be ideally cleared, and is indeed cleared once the system trajectory is steered to the safe set) and the side effects of the drug (including its ability to foster the emergence of resistance), which are assumed to be proportional to the drug concentration. We formally establish the existence of an optimal solution to the considered problem (Theorem 4.1). We analyse necessary conditions for optimality based on the Pontryagin minimum principle in Section 4.1 and singular controls in Section 4.2, following in the footsteps of [38, 39]. Moreover, in Section 4.3 we propose an alternative finite-horizon model predictive control approach [45] for treatment design. Finally, Section 5 presents numerical simulations showcasing both the solution to the optimal control problem and the alternative MPC approach, with system parameters taken from the biological literature [16], across various scenarios that include either two drugs or three drugs and several different initial conditions; the simulations successfully validate our theoretical results and demonstrate the effectiveness of the control approach in clearing the infection.

## Single-Antibiotic AMR Model and Its Analysis

We analyse here a compartmental dynamic model that describes the evolution of antimicrobial resistance in bacteria during treatment with a single antibiotic. Let *s*(*t*) denote the population size of susceptible bacteria, *r*(*t*) the population size of resistant bacteria, $b(t) = r(t) + s(t)$ the total bacterial population size, and *u*(*t*) the antibiotic concentration, constrained by $0 \le u(t) \le {\bar{u}}$, where ${\bar{u}}$ is the maximum tolerated concentration. The time evolution of the two populations is described by the ordinary-differential-equation system

1 $$\begin{aligned} \begin{array}{ll} \dot{s}(t) = \alpha s(t) - \frac{\beta }{1+ \gamma b(t)} s(t) - u(t) s(t) \Big (\eta + \nu \frac{r(t)}{b(t)}\Big ) - \mu u(t) s(t) \\ \dot{r}(t) = \alpha r(t) - \frac{\beta }{1+ \gamma b(t)} r(t) + u(t) s(t) \Big (\eta + \nu \frac{r(t)}{b(t)}\Big ), \end{array} \end{aligned}$$

where:

1. The net **growth** rate $\alpha >0$ in the absence of antibiotics and immune response is assumed to be the same for both susceptible and resistant bacteria.
2. The response function of the host **immune system**, phenomenologically modelled as in [16, 27, 53] under the assumption that the populations of phagocytes and bacteria are well mixed, is characterised by coefficients $\beta >0$ and $\gamma >0$, where $\beta$ can be interpreted as the maximal killing rate of bacteria due to activated phagocytes and $\frac{1}{\gamma }$ is the number of activated phagocytes, assumed to be constant; susceptible and resistant bacteria are assumed to be equally vulnerable to the action of the immune system.
3. The function $\phi \Big (\frac{r(t)}{b(t)}\Big ){:}{=} \eta + \nu \frac{r(t)}{b(t)} \in [\eta , \eta +\nu ]$ describes the rate at which susceptible bacteria turn into resistant bacteria, through two different mechanisms: mutations and horizontal gene transfer. **Mutations** cause a fraction of susceptible bacteria to become resistant, with a flow that we assume is proportional to *s* through the coefficient $\eta >0$. **Horizontal gene transfer** allows resistance to be acquired through the transfer of genetic material between bacteria, and is modelled as a contagion phenomenon: susceptible bacteria can be “infected”, and thus develop resistance, when they interact with resistant bacteria, so that the flow is proportional to $\frac{s r}{b}$ through the coefficient $\nu >0$. The onset of resistance is assumed to be primarily caused by antibiotic selective pressure, and thus occur only in the presence of antibiotics, whose concentration modulates the rate function $\phi$; spontaneous mutations occurring without antibiotic usage are considered negligible, since they are unlikely to occur in the considered time horizon, and are implicitly accounted for by the initial presence of resistant bacteria (i.e., by the initial conditions of the model). We also neglect spontaneous mutations turning resistant bacteria into susceptible bacteria, which amounts to considering a worst-case scenario.
4. In the term capturing the effect of the **antibiotic**, $\mu >0$ is the killing rate of susceptible bacteria due to the antibiotic therapy.

### Remark 2.1

The conversion of susceptible bacteria into resistant bacteria may be a slow or a relatively fast process. Every time a bacterial cell divides, there is a non-negligible probability of a resistance-inducing mutation. The rate at which cell division occurs can largely vary, depending on the bacterial species and on the environmental conditions: common pathogenic bacteria can divide every 20-40 minutes in laboratory conditions (e.g., 20 minutes for the bacterium *Escherichia coli*) and every few hours (from 1-2 up to about 24 hours) *in vivo* or in the wild, while an extreme case is that of *Syntrophobacter fumaroxidans*, which only doubles in the laboratory every 140 hours [24]. Also, the phenomenon of plasmid transfer may be subject to delay. This justifies resorting, in some scenarios of interest, to delay-differential-equation models that incorporate an explicit delay in the process turning sensitive bacteria into resistant bacteria [15]; see also [23, 34]. This is an interesting direction for future work.

**Fig. 1:**
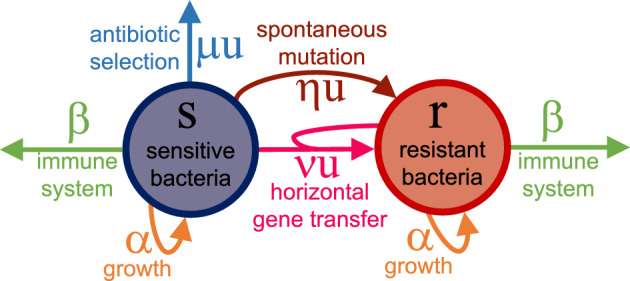
Visual representation of the compartmental model (1) describing the evolution of susceptible (*s*) and resistant (*r*) bacterial populations in the presence of a single antibiotic with concentration *u*.

The schematic capturing the considered interactions is shown in Figure 1.

For the system to be biologically meaningful, the bacterial population size cannot become negative throughout the system evolution. This is indeed guaranteed, because the system in (1) is positive: the nonnegative orthant $\left\{ (s,r) :s \ge 0,~ r \ge 0\right\}$ is an invariant set for the system (in fact, when $s=0$ it is $\dot{s} = 0$ and when $r=0$ it is $\dot{r} \ge 0$).

We aim at driving the system towards the pathogen-free state $s=r=0$ (hence, $b=0$), which is an equilibrium of system (1) for all *u*.

The killing rate $\frac{\beta }{1+ \gamma b(t)}$ due to the immune system is a decreasing function of *b*, which converges to zero as $b \rightarrow \infty$. We assume that the maximal killing rate $\beta$ is larger that the maximal net growth rate $\alpha$ of bacteria.

### Assumption 1

The maximal killing rate $\beta$ and the maximal net growth rate $\alpha$ of bacteria satisfy the inequality $\beta > \alpha$.

As we will see in the following, Assumption 1 is needed to ensure that the equilibrium at the origin of system (1), corresponding to a pathogen-free state, is asymptotically stable in the absence of treatment.

### Assumption 2

The maximum tolerated antibiotic concentration ${\bar{u}}$ satisfies the inequality ${\bar{u}} > \frac{\alpha }{\mu }$.

Assumption 2 guarantees that, in system (1), $\dot{s} < 0$ when $u = {\bar{u}}$: the considered antibiotic, at tolerated concentrations, can suppress the growth of susceptible bacteria (consistently with the concept of susceptible bacteria).

**Fig. 2:**
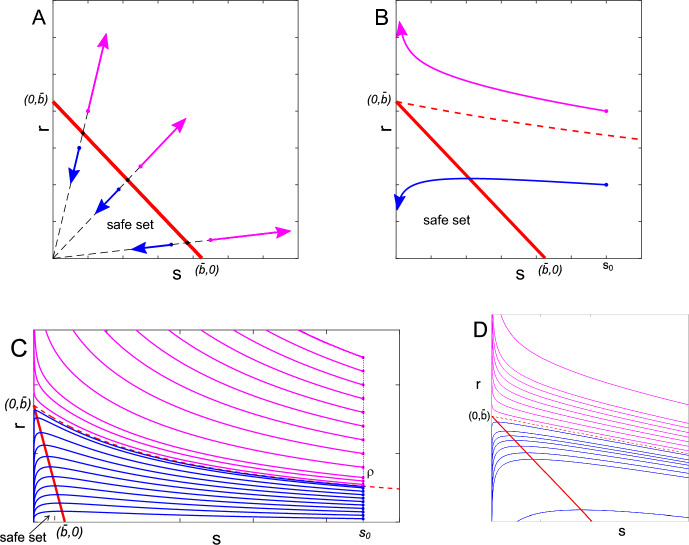
Simulated evolution of system (1) with parameters $\alpha = 0.27$, $\beta = 0.5$, $\gamma = 1$, $\nu = 0.0001$, $\eta = 0.0002$, $\mu = 0.1786$, in the absence of treatment, $u \equiv 0$ (**A**) and in the presence of constant treatment $u \equiv {\bar{u}} = 2 \frac{\alpha }{\mu }$ (**B-C-D**). The threshold line $s+r = {\bar{b}} \approx 0.8519$, with ${\bar{b}}$ defined in (3), is in red; the safe set, from which the trajectories converge to the origin even with $u \equiv 0$, is given by nonnegative states (*s*, *r*) for which $s+r < {\bar{b}}$. The example trajectories in blue converge to the origin, those in magenta diverge. **A:** when $u \equiv 0$, the system trajectory emanating from $(s_0,r_0)$ evolves along a line through the origin; it converges to the origin if $s_0+r_0 < {\bar{b}}$ and it diverges if $s_0+r_0 > {\bar{b}}$, while it is at an equilibrium if $s_0+r_0 = {\bar{b}}$. **B-C-D:** the presence of antibiotics expands the set of points from which the trajectories converge to the origin; the boundary of such a set is given by the red dashed curve, which is a function $r=\theta (s)$ with ${\bar{b}} = \theta (0)$. **B:** Given $s_0=1.2$, the trajectory emanating from $(s_0,\rho )$ enters the safe set and thus converges to the origin when $\rho =0.4$, while it diverges ($s \rightarrow 0$, while $r \rightarrow \infty$) when $\rho =0.8$. **C:** Given $s_0=9$, the trajectory emanating from $(s_0,\rho )$ converges to $(0, {\bar{b}})$ when $\rho = r_0 \approx 0.26446798$ (red dashed curve), enters the safe set and thus converges to the origin when $\rho < r_0$, and diverges when $\rho > r_0$. **D:** Same as panel C, in a neighbourhood of $(0,{\bar{b}})$, with $s \in [0,0.2]$ and $r \in [0.75,0.95]$.

We consider some fundamental properties of the second-order model (1), so as to lay the foundations for addressing more complex scenarios later on. We focus in particular on two cases that offer important insight: the evolution in the absence of antibiotics, $u \equiv 0$ (see Figure 2A), and the evolution in the presence of a single antibiotic with constant $u \equiv {\bar{u}}$ (see Figure 2B and C).

When $u \equiv 0$, adding the two equations of system (1) shows that the total bacterial population size evolves as

2 $$\begin{aligned} \dot{b}(t) = \alpha b(t) - \frac{\beta }{1+ \gamma b(t)} b(t) = \psi (b(t)) b(t), \end{aligned}$$

where $$\psi (b){:}{=} \alpha - \frac{\beta }{1+\gamma b}$$.

### Proposition 2.1

Under Assumption 1, system (2) admits two equilibria, the asymptotically stable equilibrium $b=0$ and the unstable equilibrium $b = {\bar{b}}$, where

3 $$\begin{aligned} {\bar{b}} {:}{=} \frac{1}{\gamma } \left( \frac{\beta }{\alpha }-1\right) . \end{aligned}$$

Trajectories emanating from an initial condition $b(0) > {\bar{b}}$ diverge, while trajectories emanating from an initial condition $0 \le b(0) < {\bar{b}}$ converge to zero.

### Proof

The equilibrium condition $\dot{b} = 0$ is satisfied when $\alpha \gamma b^2 + (\alpha -\beta )b=0$, which has the two solutions $b=0$ and $b = {\bar{b}}>0$. When $b > {\bar{b}}$, it is $\dot{b}>0$, and hence *b* diverges, which proves instability of the equilibrium $b={\bar{b}}$. When $0< b < {\bar{b}}$, it is $\dot{b}<0$, and hence *b* converges to 0, which proves asymptotic stability of the equilibrium $b=0$, with domain of attraction $[0, {\bar{b}})$. $\square$

### Proposition 2.2

The equilibrium (0, 0) of system (1) is asymptotically stable when $u \equiv 0$ if and only if Assumption 1 holds.

### Proof

When $s=r=0$, $\dot{s} = \dot{r} = 0$ regardless of *u*, hence (0, 0) is indeed an equilibrium for system (1). In particular, when $u \equiv 0$, the system Jacobian is

$$J_{(s,r)}=\begin{bmatrix} \alpha - \frac{\beta (1+\gamma r)}{(1+\gamma (s+r))^2} &  \frac{\beta \gamma s}{(1+\gamma (s+r))^2} \\ \frac{\beta \gamma r}{(1+\gamma (s+r))^2} &  \alpha - \frac{\beta (1+\gamma s)}{(1+\gamma (s+r))^2} \end{bmatrix}.$$

Computing the Jacobian at the equilibrium at the origin yields

$$J_{(0,0)}=\begin{bmatrix} \alpha -\beta &  0\\ 0 &  \alpha -\beta \end{bmatrix},$$

and hence (0, 0) is asymptotically stable if and only if $\alpha -\beta <0$, which corresponds to Assumption 1. In fact, when $\alpha >\beta$ the Jacobian has two positive eigenvalues, while when $\alpha =\beta$ (and hence $\det (J_{(0,0)})=0$) it is $\dot{b} = \frac{\beta \gamma b^2}{1 + \gamma b} >0$ for all $b > 0$, and therefore the equilibrium at zero is unstable. $\square$

### Theorem 2.1

Consider system (1) with $u \equiv 0$, under Assumption 1. Every system trajectory emanating from the initial condition (*s*(0), *r*(0)) evolves along a line through the origin. Given the value ${\bar{b}}$ defined in (3), $s+r = {\bar{b}}$ is a threshold line in the *s*-*r* plane such that if $s(0)+r(0) < {\bar{b}}$, the trajectory converges to the origin, while if $s(0)+r(0) > {\bar{b}}$, the trajectory diverges. The values $({\bar{s}}, {\bar{r}})$ for which ${\bar{s}} + {\bar{r}} = {\bar{b}}$ are unstable equilibria for the system, while the asymptotically stable equilibrium (0, 0) has domain of attraction

4 $$\begin{aligned} {\mathcal {S}} = \left\{ (s,r):s+r < {\bar{b}}, ~s \ge 0,~r \ge 0 \right\} , \end{aligned}$$

which we denote as *safe set*.

### Proof

When $u \equiv 0$, we can write $\dot{s} = \psi (b) s$, $\dot{r} = \psi (b) r$ and $\dot{b} = \psi (b) b$, where $\psi (b) = \alpha - \frac{\beta }{1+\gamma b}$. If $b(0)=s(0)+r(0)=0$, i.e. $s(0)=r(0)=0$, we are at an equilibrium as per Proposition 2.2. If $b(0) \ne 0$, the fractions of susceptible and resistant bacteria in the population remain constant: $\frac{d}{dt}{(\frac{s}{b})}=\frac{\dot{s} b - \dot{b} s}{b^2}=0$ and $\frac{d}{dt}{(\frac{r}{b})}=\frac{\dot{r} b - \dot{b} r}{b^2}=0$; hence, $s(t)=\frac{s(0)}{b(0)} b(t)$ and $r(t)=\frac{r(0)}{b(0)} b(t)$. If $s(0)=0$, $s \equiv 0$, while $r=b$ evolves as in Proposition 2.1. If $r(0)=0$, $r \equiv 0$, while $s=b$ evolves as in Proposition 2.1. If both $s(0) \ne 0$ and $r(0) \ne 0$, the trajectory evolves along a line $r(t) = \frac{r(0)}{s(0)} s(t)$; as a consequence, both *s* and *r* converge to zero, remain constant or diverge whenever *b* does, and the result thus follows from Propositions 2.1 and 2.2. $\square$

The *safe set* ${\mathcal {S}}$ is the set of all points in the *s*-*r* plane from which the trajectories of system (1) converge to zero when $u \equiv 0$. Convergence to zero of all trajectories with initial conditions in the safe set is also ensured for any *u*, and the closure $\bar{{\mathcal {S}}}$ of ${\mathcal {S}}$ is an invariant set for system (1) regardless of *u*.

### Proposition 2.3

Consider system (1) under Assumption 1. All the system trajectories emanating from the safe set ${\mathcal {S}}$ defined in (4) converge to the origin regardless of *u*. Moreover, the set $\bar{{\mathcal {S}}} = \left\{ (s,r):s+r \le {\bar{b}}, ~s \ge 0,~r \ge 0 \right\}$ is positively invariant for the system, regardless of *u*.

### Proof

Adding the two equations in (1) yields $\dot{b}(t) = \psi (b(t)) b(t)-\mu u(t)s(t)$. Whenever $s+r = b \le {\bar{b}}$, it is $\dot{b} \le 0$ and, since the system is positive, invariance of $\bar{{\mathcal {S}}}$ is ensured; whenever $0< s+r = b < {\bar{b}}$, it is $\dot{b} < 0$, which entails convergence to the origin from any point in ${\mathcal {S}}$. $\square$

We now consider the case in which the antibiotic is present at a constant concentration $u \equiv {\bar{u}}$.

### Proposition 2.4

System (1) with $u \equiv {\bar{u}}$, under Assumptions 1 and 2, admits two equilibria, the asymptotically stable equilibrium (0, 0) and the unstable equilibrium $(0, {\bar{b}})$. In particular, $(0,{\bar{b}})$ is a hyperbolic equilibrium point with one positive eigenvalue and one negative eigenvalue.

### Proof

When $u \equiv {\bar{u}}$ is constant, $\dot{s}=0$ if and only if $s=0$; then, $\dot{r} = \psi (r)r=0$ if and only if either $r=0$ or $r={\bar{b}}$, by Proposition 2.1. The system Jacobian

$$J_{(s,r)}=\begin{bmatrix} \alpha - \frac{\beta (1+\gamma r)}{(1+\gamma (s+r))^2}-{\bar{u}} (\mu +\eta +\frac{\nu r}{s+r})+{\bar{u}} \frac{\nu s r}{(s+r)^2} &  \frac{\beta \gamma s}{(1+\gamma (s+r))^2}-{\bar{u}} \frac{\nu s^2}{(s+r)^2} \\ \frac{\beta \gamma r}{(1+\gamma (s+r))^2}+{\bar{u}} (\eta +\frac{\nu r}{s+r})-{\bar{u}} \frac{\nu s r}{(s+r)^2} &  \alpha - \frac{\beta (1+\gamma s)}{(1+\gamma (s+r))^2}+{\bar{u}} \frac{\nu s^2}{(s+r)^2} \end{bmatrix}$$

computed at the origin yields

$$J_{(0,0)}=\begin{bmatrix} \alpha -\beta -{\bar{u}} (\mu +\eta ) &  0\\ {\bar{u}} \eta &  \alpha -\beta \end{bmatrix},$$

hence the equilibrium at the origin is asymptotically stable by Assumption 1. Computing the Jacobian at $(0,{\bar{b}})$ yields

$$J_{(0,{\bar{b}})}=\begin{bmatrix} \alpha - \frac{\beta }{1+\gamma {\bar{b}}}-{\bar{u}} (\mu +\eta +\nu ) &  0\\ \frac{\beta \gamma {\bar{b}}}{(1+\gamma {\bar{b}})^2}+{\bar{u}} (\eta +\nu ) &  \alpha -\frac{\beta }{(1+\gamma {\bar{b}})^2} \end{bmatrix},$$

whose eigenvalues are $\lambda _1 = -{\bar{u}} (\mu +\eta +\nu )<0$, because $\alpha - \frac{\beta }{1+\gamma {\bar{b}}}=\psi ({\bar{b}})=0$, and $\lambda _2= \alpha -\frac{\beta }{(1+\gamma {\bar{b}})^2} = \alpha (1-\frac{\alpha }{\beta })>0$ in view of Assumption 1. Therefore, the hyperbolic equilibrium $(0,{\bar{b}})$ is unstable. $\square$

### Proposition 2.5

The trajectories of system (1) with $u \equiv {\bar{u}}$, under Assumptions 1 and 2, that emanate from initial condition $(s(0),r(0)) \in \{(s,r) :s,r\ge 0, \, r \ge {\bar{b}}\} \setminus (0,{\bar{b}})$ diverge, i.e., $\lim _{t \rightarrow \infty } r(t)=\infty$.

### Proof

If $s(0)=0$, then $\dot{s} = 0$ for all $t \ge 0$ and $\dot{r} = \psi (r) r$, since $r \equiv b$. Therefore, *r* diverges if $r(0) > {\bar{b}}$, as per Proposition 2.1. If $s(0)>0$ and $r(0) \ge {\bar{b}}$, it is $\dot{r} = \psi (b)r + {\bar{u}} s (\eta + \nu \frac{r}{b})) \ge {\bar{u}} s (\eta + \nu \frac{r}{b})) >0$ at $t=0$. Then, for small $t_1>0$, $r(t_1) \ge {\bar{b}} +\epsilon$ and hence $\dot{r} = \psi (b)r + {\bar{u}} s (\eta + \nu \frac{r}{b})) \ge \psi ({\bar{b}} + \epsilon ) r > 0$ for all $t\ge t_1$. Therefore, the trajectory diverges, that is $r(t) \ge r(t_1) e^{\sigma (t-t_1)}$ for all $t\ge t_1$, where $\sigma =\psi ({\bar{b}}+\epsilon )>0$. $\square$

### Theorem 2.2

Given system (1) with $u \equiv {\bar{u}}$, under Assumptions 1 and 2, consider a trajectory emanating from the initial condition (*s*(0), *r*(0)). There exists a threshold function $r=\theta (s)$, depending on ${\bar{u}}$, with ${\bar{b}}=\theta (0)$, such that the trajectory converges to the origin if $r(0) < \theta (s(0))$, and diverges if $r(0) > \theta (s(0))$. If $r(0) = \theta (s(0))$, the trajectory evolves along the separatrix curve $r(t) = \theta (s(t))$ and converges to the point $(0,{\bar{b}})$. Hence, the set

5 $$\begin{aligned} {\mathcal {D}}{:}{=}\left\{ (s,r):r < \theta (s), ~s \ge 0,~r \ge 0 \right\} \end{aligned}$$

is the domain of attraction of the equilibrium at the origin for the dynamics (1) with $u \equiv {\bar{u}}$.

### Proof

We conduct the proof with the support of the visualisation in Figures 2B and 2C, where the threshold function $\theta (s)$ is shown as a red dashed separatrix curve. Consider the initial condition $(s_0,\rho )$, where $s_0$ is fixed and $\rho >0$ is a parameter, as shown in Figure 2C. Since the system trajectories in the *s*-*r* plane do not intersect (if they did, this would violate uniqueness of the solution), given the trajectories $(s_1(t),r_1(t))$ emanating from $(s_0,\rho _1)$ and $(s_2(t),r_2(t))$ emanating from $(s_0,\rho _2)$, if $\rho _1 > \rho _2$, then $r_1(t)>r_2(t)$ for all $t \ge 0$.

When $s_0=0$, $s \equiv 0$ (since $\dot{s} = 0$) and hence $r \equiv b$. Since $\dot{r} = \psi (r) r$, *r* converges to the origin if $\rho < {\bar{b}}$ and diverges if $\rho > {\bar{b}}$, while $\rho = {\bar{b}}$ is an equilibrium (see Proposition 2.1 and Proposition 2.5). Hence, $\theta (0)={\bar{b}}$.

Also, since $\mu {\bar{u}}>\alpha$ by Assumption 2, $s > 0$ implies $\dot{s}< (\alpha - \mu {\bar{u}}) s < 0$, and hence $\lim _{t \rightarrow \infty } s(t)=0$.

By Proposition 2.4, the equilibrium $(0, {\bar{b}})$ is hyperbolic with a positive and a negative eigenvalue; therefore, the Stable Manifold Theorem (see, e.g., [52, pp. 107-108]) implies the existence of a local one-dimensional stable manifold (while the one-dimensional unstable manifold is the *r*-axis). Consider a point $(s_{M,0},r_{M,0})$ on the stable manifold and the solution $(s_M(t),r_M(t))$ emanating from that point, also extended backward in time. Since, for any $\delta >0$, $s > \delta$ implies $\dot{s}< (\alpha - \mu {\bar{u}}) \delta < 0$, the set ${\mathcal {M}}=\{(s_M(t),r_M(t)), t \in {\mathbb {R}}\}$ intersects each vertical line of the form $s=c_s>0$ exactly once, thereby inducing a continuous function $\theta$ defined for ${\hat{s}} \in [0,\infty )$ such that $\theta (0)={\bar{b}}$ and $\theta ({\hat{s}})$ is the unique value of *r* at the intersection of the set ${\mathcal {M}}$ with $\{s = {\hat{s}}\}$. By (1) and the implicit function theorem, $\theta ({\hat{s}})$ is continuously differentiable for all ${\hat{s}}>0$.

Since the one-dimensional stable manifold is locally a graph of a function, no other trajectory can converge to $(0, {\bar{b}})$, and hence the separatrix $r = \theta (s)$, $s\in [0,\infty )$, due to the uniqueness of the solution, divides the nonnegative orthant into two regions.

Any trajectory emanating from a point with $r_0<\theta (s_0)$ evolves, for all future times, in a bounded set with $0 \le s \le s_0$ and $0 \le r < \theta (s)$, because trajectories cannot intersect. Since (1) is a planar autonomous system, the Poincaré-Bendixson theorem applies. Being $\dot{s} < 0$ for all $s>0$, the trajectory cannot converge to a periodic orbit, and homoclinic or heteroclinic orbits connecting the two equilibria cannot exist (also in view of the analysis of the dynamics with $s \equiv 0$ above). Thus, the trajectory must converge to an equilibrium point, which must be the origin (convergence to $(0, {\bar{b}})$ would contradict one-dimensionality of the stable manifold). Hence, all trajectories emanating from points below the separatrix, with $r<\theta (s)$, converge to the origin.

Conversely, all trajectories emanating from points above the separatrix, with $r>\theta (s)$, diverge; in fact, they cannot converge to either of the two equilibria, or to periodic orbits, or to homoclinic or heteroclinic orbits, as discussed above, and hence their *r*-coordinate cannot be bounded. Therefore, in view of Proposition 2.5, it must be $\lim _{t\rightarrow \infty } r(t) = \infty$.

As a consequence, the domain of attraction of the equilibrium at the origin for system (1) with $u \equiv {\bar{u}}$ is given by (5). $\square$

In light of Proposition 2.3, once the trajectories reach the safe set ${\mathcal {S}}$ defined in (4), they then converge to the origin regardless of the chosen *u*(*t*), because the effect of the immune system is sufficient to suppress bacterial growth. Hence, the goal of an antibiotic therapy is to steer the system trajectories into ${\mathcal {S}}$. This is the problem we consider.

### Problem 2.1

Find a therapy (in terms of drug concentration) such that the trajectories of the system reach the safe set (i.e., the domain of attraction of the equilibrium at the origin for the dynamics with $u \equiv 0$) in finite time.

Under Assumption 2, in view of Theorem 2.2, for any initial condition $(s_0,r_0) \in {\mathcal {D}}$ in (5), with $r_0 < \theta (s_0)$, the problem admits a solution, while no solution to the problem exists for initial conditions $(s_0,r_0) \not \in {\mathcal {D}}$, with $r_0 \ge \theta (s_0)$, assuming a maximum tolerated concentration ${\bar{u}}$.

In the case of the two-dimensional model (1), a possible solution is to maintain a constant maximal antibiotic concentration ${\bar{u}}$ until the system trajectories are driven within the safe set; at that point, the therapy can be stopped. This ensures that the safe set is reached in minimum time. Possibly, a smaller antibiotic concentration is also able to drive the system trajectories to the safe set, although over a longer time horizon.

The existence of many different choices of *u*(*t*) that drive the system trajectories within the safe set leaves room for optimisation according to different criteria, depending on the chosen cost functional. For instance, one may seek the smallest possible drug concentration such that the system trajectories reach the safe set within a prescribed horizon, so as to minimise side effects.

The problem becomes significantly more complex when considering multiple antibiotics and the presence of multidrug resistance.

Problem formulations analogous to Problem 2.1 have already been considered for bacterial infections [3, 4, 16], as well as for cancer treatment under interactions with the immune system. In particular, [38, 39] consider a model of immunological activity during cancer growth, and formulate, analyse and solve an optimal control problem to design treatment protocols (for chemotherapy in [39] and for combined chemotherapy and immunotherapy in [38]) that steer the system from an initial condition in the malignant growth region of the state space (the domain of attraction of the malignant equilibrium) into the benign region (the domain of attraction of the benign equilibrium), by choosing a cost function based on the stable manifold of the saddle point, which describes the separatrix between the regions of benign and malignant growth.

### Effects of Logistic Growth

Since in (1) we consider a purely linear net growth and we neglect a possible logistic term capturing limitation of resources, the total bacterial population size may grow indefinitely. Although this is biologically unrealistic, we make this modelling choice for simplicity as it does not affect our goals. In fact, our objective is to avoid any outcome that differs from convergence to the pathogen-free equilibrium, regardless of whether the total bacterial population converges to a finite non-zero equilibrium state, upper bounded by the carrying capacity (which is what would happen in the presence of a logistic growth term), or to infinity (which is what happens in its absence). In particular, considering a logistic term would modify (1) into

6 $$\begin{aligned} \begin{array}{ll} \dot{s}(t) = \alpha s(t) \Big (1- \dfrac{b(t)}{N}\Big ) - \dfrac{\beta }{1+ \gamma b(t)} s(t) - u(t) s(t) \Big (\eta + \nu \dfrac{r(t)}{b(t)}\Big ) - \mu u(t) s(t), \\ \dot{r}(t) = \alpha r(t) \Big (1- \dfrac{b(t)}{N}\Big ) - \dfrac{\beta }{1+ \gamma b(t)} r(t) + u(t) s(t) \Big (\eta + \nu \dfrac{r(t)}{b(t)}\Big ), \end{array} \end{aligned}$$

where $N>0$ is the carrying capacity. Also system (6) is positive and, when $u \equiv 0$, the total bacterial population size evolves as

7 $$\begin{aligned} \dot{b}(t)=\psi _N(b(t)) b(t), \end{aligned}$$

where

$$\begin{aligned} \psi _N(b){:}{=}\alpha \Big (1-\frac{b}{N}\Big )-\frac{\beta }{1+\gamma b} = \psi (b) - \alpha \frac{b}{N}. \end{aligned}$$

While $b=0$ is always an equilibrium, system (7) may admit more equilibria depending on the discriminant $\Delta = \alpha ^2\Big (\gamma -\frac{1}{N}\Big )^2-\frac{4\alpha \gamma }{N}(\beta -\alpha )$ of the quadratic equation $\psi _N(b)=0$ [3]. If $\Delta <0$, then $\psi _N(b)<0$ for all $b>0$: the origin is the only equilibrium and it is asymptotically stable, since $\dot{b}<0$ for all $b>0$; all trajectories converge to the origin even without therapy, whence the safe set for system (6) is the whole nonnegative orthant. If $\Delta =0$, there is a single nonzero equilibrium ${\bar{b}}$ and, since $\dot{b} <0$ for both $0< b < {\bar{b}}$ and $b > {\bar{b}}$, the equilibrium at the origin is asympotically stable while ${\bar{b}}$ is unstable; hence, system (6) has equilibria $(0, {\bar{b}})$, unstable, and (0, 0), asymptotically stable, with safe set ${\mathcal {S}}_N = \left\{ (s,r):s+r < {\bar{b}}, ~s \ge 0,~r \ge 0 \right\}$. If $\Delta >0$, there are two nonzero equilibria ${\bar{b}}_1< {\bar{b}}_2 < N$: the equilibria at zero and ${\bar{b}}_2$ are asymptotically stable, while ${\bar{b}}_1$ is unstable, because $\dot{b} < 0$ for $0< b < {\bar{b}}_1$, while $\dot{b} > 0$ for ${\bar{b}}_1< b < {\bar{b}}_2$ and $\dot{b} < 0$ for $b > {\bar{b}}_2$; hence, system (6) has equilibria $(0, {\bar{b}}_2)$, asymptotically stable, $(0, {\bar{b}}_1)$, unstable and hyperbolic, and (0, 0), asymptotically stable, with safe set ${\mathcal {S}}_N = \left\{ (s,r):s+r < {\bar{b}}_1, ~s \ge 0,~r \ge 0 \right\}$. Hence, the logistic term changes the model dynamics by preventing the divergence of trajectories that occurs in (1) and replacing it, for suitable parameter values, with convergence to a finite equilibrium $(0, {\bar{b}}_2)$; however, this change in behaviour only affects scenarios in which no viable therapy can drive the system trajectories to the safe set, and hence Problem 2.1 admits no solution.

Including the logistic growth also reshapes the threshold function for constant $u \equiv {\bar{u}}$. If $\Delta <0$, there is no separatrix, as all initial conditions in the nonnegative orthant converge to zero. If $\Delta =0$, there is a separatrix curve $r=\theta _N(s)$, with $\theta _N(0)={\bar{b}}$, such that all trajectories emanating from points with $r \ge \theta _N(s)$ converge to $(0,{\bar{b}})$, while all trajectories emanating from points with $r < \theta _N(s)$ converge to the origin. Again by the Stable Manifold Theorem, if $\Delta >0$, the hyperbolic equilibrium $(0, {\bar{b}}_1)$ has a one-dimensional stable manifold corresponding to a curve $r=\theta _N(s)$, with $\theta _N(0)={\bar{b}}_1$, which partitions the state space as follows: all trajectories emanating from points with $r > \theta _N(s)$ converge to $(0,{\bar{b}}_2)$, all trajectories emanating from points on the separatrix $r = \theta _N(s)$ converge to $(0,{\bar{b}}_1)$, while all trajectories emanating from points with $r < \theta _N(s)$ converge to the origin.

Concerning the effect on the solution to Problem 2.1 and on optimal therapies, neglecting the logistic term yields a worst-case scenario. In fact, since $\psi _N(b) \le \psi (b)$ for all $b\ge 0$, by the comparison principle, the total bacterial growth in the logistic model (6) is never larger than in model (1), since, for all $u(t) \ge 0$, $\psi _N(b)b-\mu u s \le \psi (b)b-\mu u s$. The equality only holds if $b=0$. Also, since $\psi _N(0)=\psi (0)=\alpha -\beta <0$ and $\psi _N(b) < \psi (b)$ for all $b>0$, the zeros of $\psi _N(b)$ (if any) occur at a value of *b* that is larger than the zero of $\psi (b)$, meaning that the safe set expands (when $\Delta <0$, it is the whole nonnegative orthant; when $\Delta \ge 0$, the separatrix shifts upward). Also, by the comparison principle, if the trajectory emanating from a given initial condition converges to the origin with model (1), it also converges to the origin with the logistic model (6); and trajectories from additional initial conditions may converge to zero under the dynamics (6), because the total bacterial growth is stricly smaller as long as $b>0$. Hence, the domain of attraction of the equilibrium at the origin in the presence of a nonzero drug concentration *u*(*t*) expands under logistic growth: clearing the infection becomes easier, *ceteris paribus*, and the required optimal drug concentration cannot increase (if $\Delta < 0$, infection clearance is even achieved without therapy). For $N\rightarrow \infty$, the dynamics of system (6) converge to those of system (1); the larger *N* is, the farther from the origin it effects are perceived, while Problem 2.1 only admits a solution for initial conditions that are sufficiently close to the equilibrium at the origin, so that the corresponding trajectories can be steered to the safe set.

## Multiple-Antibiotic AMR Model

We first model bacterial population dynamics in the scenario with two antibiotics, which lays the foundation for the subsequent generalisation to the case of *m* antibiotics.

### Two Antibiotics

When two different antibiotics *A* and *B* are administered, having concentration $u_A$ and $u_B$ respectively, bacterial populations can arise that are resistant to antibiotic *A* (with population size $r_A$), to antibiotic *B* (with population size $r_B$) and to both antibiotics (with population size $r_{AB}$). The total bacterial population size is

8 $$\begin{aligned} b(t)=s(t)+r_A(t)+r_B(t)+r_{AB}(t). \end{aligned}$$

Let $\mu _A$ and $\mu _B$ represent the killing rate of susceptible bacteria *s* due to antibiotics *A* and *B*, respectively, while $\mu _{A,B}$ denotes the killing rate of $r_B$ due to antibiotic *A* and $\mu _{B,A}$ the killing rate of $r_A$ due to antibiotic *B*.

**Fig. 3:**
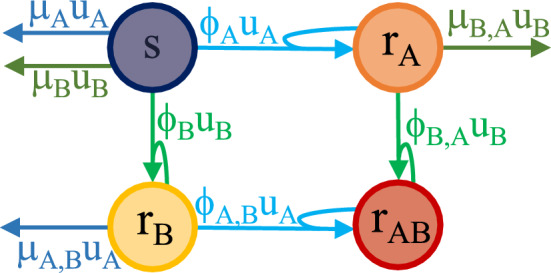
Antibiotic killing rates $\mu _X$ and antibiotic-induced rates $\phi _X$ of resistance onset for the case of two antibiotics with concentrations $u_A$ and $u_B$. Interactions that are present when antibiotic *A* (respectively, *B*) is administered are in shades of blue (respectively, green).

The functions capturing the rate of antibiotic-induced emergence of resistance, due to both mutations and horizontal gene transfer, have the form $\phi _X \Big (\frac{r_Y(t)}{b(t)}\Big ){:}{=} \eta _X + \nu _X \frac{r_Y(t)}{b(t)}$. In particular, when $u_A \ne 0$, $\phi _{A}(r_A/b)$ turns susceptible bacteria into bacteria that are resistant to *A* and $\phi _{A,B}(r_{AB}/b)$ turns bacteria that are resistant to *B* into bacteria that are resistant to both antibiotics; when $u_B \ne 0$, $\phi _{B}(r_B/b)$ turns susceptible bacteria into bacteria that are resistant to *B* and $\phi _{B,A}(r_{AB}/b)$ turns bacteria that are resistant to *A* into bacteria that are resistant to both antibiotics. These interactions are visualised in Figure 3.

Denoting by $\psi (b(t)){:}{=} \alpha - \frac{\beta }{1+\gamma b(t)}$, the resulting model is

9 $$\begin{aligned} \begin{array}{ll} \dot{s} = \psi (b) s - [\mu _A + \phi _A(r_A/b)] u_A s - [\mu _B + \phi _B(r_B/b)] u_B s \\ \dot{r}_A = \psi (b) r_A + \phi _A(r_A/b) u_A s - [\mu _{B,A} + \phi _{B,A}(r_{AB}/b)] u_B r_A \\ \dot{r}_B = \psi (b) r_B + \phi _B(r_B/b) u_B s - [\mu _{A,B} + \phi _{A,B}(r_{AB}/b)] u_A r_B \\ \dot{r}_{AB} = \psi (b) r_{AB} + \phi _{B,A}(r_{AB}/b) u_B r_A+ \phi _{A,B}(r_{AB}/b) u_A r_B, \end{array} \end{aligned}$$

where for simplicity we have dropped the explicit indication of time dependency for the state variables, the total bacterial population and the drug concentrations.

Although the model complexity significantly increases when two antibiotics are considered, the problem of finding a suitable therapy (now corresponding to drug concentrations $u_A(t)$ and $u_B(t)$) that steers the system trajectories into the safe set is still of interest. In fact, the same properties outlined in Proposition 2.3 for system (1) still hold in this more complex case, by redefining the safe set as follows.

#### Proposition 3.1

Consider system (9) where, in function $\psi (b)$, $\beta > \alpha$. The safe set

10 $$\begin{aligned} {\mathcal {S}} = \left\{ (s,r_A,r_B,r_{AB}) \ge 0 :s+r_A+r_B+r_{AB} = b < {\bar{b}}\right\} , \end{aligned}$$

where ${\bar{b}}$ is defined in (3), is the domain of attraction of the equilibrium at the origin with $u_A=u_B=0$. Then, the system trajectories emanating from any point in ${\mathcal {S}}$ converge to the origin for all possible choices of $u_A \ge 0$ and $u_B \ge 0$. The closure of the safe set,

$$\begin{aligned} \bar{{\mathcal {S}}} = \left\{ (s,r_A,r_B,r_{AB}) \ge 0 :s+r_A+r_B+r_{AB} = b \le {\bar{b}}\right\} , \end{aligned}$$

is positively invariant for the system for all possible $u_A \ge 0$ and $u_B \ge 0$.

#### Proof

Summing up the equations of system (9) yields

11 $$\begin{aligned} \dot{b} = \psi (b) b - (\mu _A u_A +\mu _B u_B)s - \mu _{B,A} u_B r_A - \mu _{A,B} u_A r_B. \end{aligned}$$

Recall that $\psi ({\bar{b}})=0$, while $\psi (b)<0$ for all $0 \le b < {\bar{b}}$ since $\beta > \alpha$. If $u_A=u_B=0$, then $\dot{b} < 0$ if and only if $0< b < {\bar{b}}$, and hence convergence to zero is achieved if and only if the initial condition is in the set ${\mathcal {S}}$ defined in (10), which thus constitutes the domain of attraction of the origin with $u_A=u_B=0$. Now consider the dynamics (11) with any $u_A \ge 0$ and $u_B \ge 0$. When $b = s+r_A+r_B+r_{AB}\le {\bar{b}}$, it is $\dot{b} \le 0$, and hence *b* cannot exceed ${\bar{b}}$, which ensures invariance of $\bar{{\mathcal {S}}}$; also, convergence of trajectories to zero is guaranteed for all $b < {\bar{b}}$ (i.e., for all points in ${\mathcal {S}}$), because $\dot{b} <0$. $\square$

Therefore, we can still investigate Problem 2.1, which in this case amounts to finding suitable drug concentrations $u_A(t)$ and $u_B(t)$ such that the system trajectories reach the safe set ${\mathcal {S}}$ defined in (10) in finite time.

The model (9) can be rewritten as

12 $$\begin{aligned} \dot{x}(t) = \psi ({\textbf {1}}^\top x(t)) x(t) + [F_A(x(t)) u_A(t) +F_B(x(t)) u_B(t)] x(t) \end{aligned}$$

where the state variable is

13 $$\begin{aligned} x(t) = \begin{bmatrix} s(t)&r_A(t)&r_B(t)&r_{AB}(t) \end{bmatrix}^\top \end{aligned}$$

and $b(t)={\textbf {1}}^\top x(t)$, while matrices $F_A(x) = F_A(\frac{r_A}{b},\frac{r_{AB}}{b})$, $F_B(x) = F_B(\frac{r_B}{b},\frac{r_{AB}}{b})$ are

14 $$\begin{aligned} F_A(x)&= \begin{bmatrix} -(\mu _A+\phi _A) & ~~ 0 &  0 & ~~0 \\ \phi _A & ~~ 0 &  0 & ~~0\\ 0 & ~~ 0 &  -(\mu _{A,B}+\phi _{A,B}) &  ~~0\\ 0 & ~~ 0 &  \phi _{A,B} &  ~~0 \end{bmatrix}, \end{aligned}$$

15 $$\begin{aligned} F_B(x)&= \begin{bmatrix} -(\mu _B+\phi _B) &  0 & ~~ 0 &  ~~0\\ 0 &  -(\mu _{B,A}+\phi _{B,A})& ~~ 0 &  ~~0\\ \phi _B &  0 & ~~ 0 &  ~~0\\ 0 &  \phi _{B,A} & ~~ 0& ~~0 \end{bmatrix}; \end{aligned}$$

for simplicity, we have dropped the explicit indication of the dependency of the resistance emergence rate functions on the state: $\phi _A = \phi _A(r_A/b)$, $\phi _B = \phi _B(r_B/b)$, $\phi _{A,B} = \phi _{A,B}(r_{AB}/b)$ and $\phi _{B,A} = \phi _{B,A}(r_{AB}/b)$.

### Multiple Antibiotics

The multiple antibiotic case is conceptually simple, but involves a complex notation. When considering *m* antibiotics, we have $2^m$ bacterial populations: each population can be either susceptible or resistant to each considered antibiotic. The differential equation describing the time evolution of the generic bacterial population size *x* includes the term $\psi (b) x$, where $b={\textbf {1}}^\top x$ denotes the total bacterial population size and $\psi (b) = \alpha - \frac{\beta }{1+\gamma b}$ captures both bacterial growth and immune response. In addition, if the population is susceptible to antibiotic *j*, the equation includes a negative term of the form $\mu _j u_j x$. Terms associated with the emergence of resistance due to the different antibiotics also need to be taken into account.

**Fig. 4:**
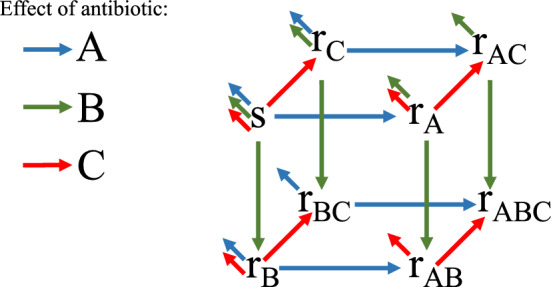
In the case of three antibiotics *A*, *B* and *C*, we associate each bacterial population with a vertex of a cube. Antibiotic-induced emergence of resistance is represented by arrows from one bacterial population to another, while antibiotic-induced suppression of bacteria is represented by arrows from one bacterial population to the external environment. Blue arrows are due to the effect of antibiotic *A*, green arrows of *B*, red arrows of *C*.

To account for the different antibiotics and resistance mechanism, we need multiple indices for the terms associated with antibiotic killing rates and antibiotic-induced resistance emergence rates. For instance, in the case of $m=3$ antibiotics *A*, *B* and *C*, the $2^m=8$ bacterial populations have size *s* (susceptible to all antibiotics), $r_A$ (resistant to antibiotic *A*), $r_B$ (resistant to antibiotic *B*), $r_C$ (resistant to antibiotic *C*), $r_{AB}$ (resistant to antibiotics *A* and *B*), $r_{AC}$ (resistant to antibiotics *A* and *C*), $r_{BC}$ (resistant to antibiotics *B* and *C*) and $r_{ABC}$ (resistant to all antibiotics) and the interactions associated with suppression due to antibiotics and with antibiotic-induced emergence of resistance are shown in Figure 4. In particular, susceptible bacteria are affected by all antibiotics with killing rates $\mu _A$, $\mu _B$, $\mu _C$, while bacteria that are resistant to antibiotic *A* are affected by antibiotic *B* with killing rate $\mu _{B,A}$ and by antibiotic *C* with killing rate $\mu _{C,A}$, bacteria that are resistant to antibiotic *B* are affected by antibiotic *A* with killing rate $\mu _{A,B}$ and by antibiotic *C* with killing rate $\mu _{C,B}$, bacteria that are resistant to antibiotic *C* are affected by antibiotic *A* with killing rate $\mu _{A,C}$ and by antibiotic *B* with killing rate $\mu _{B,C}$ (in general, $\mu _{P,Q}$ denotes the killing rate of bacteria that are resistant to *Q* due to antibiotic *P*); also bacterial populations that are resistant to two antibiotics are affected by the third antibiotic with killing rates $\mu _{A,BC}$, $\mu _{B,AC}$ and $\mu _{C,AB}$ (where $\mu _{P,QR}$ denotes the killing rate of bacteria that are resistant to both *Q* and *R* due to antibiotic *P*). Functions $\phi _X(r_Y/b)$, associated with the onset of resistance, follow the same indexing rule.

In the general case with *m* populations, we can associate each population with a vertex of an *m*-dimensional cube. The killing rate of susceptible bacteria due to antibiotic *P* is $\mu _{P}$, while $\mu _{P,QR\dots }$ denotes the killing rate of bacteria that are resistant to antibiotics *Q*, *R*, $\dots$, due to antibiotic *P*. Also, function $\phi _{P}$ denotes the rate at which susceptible bacteria acquire resistance to antibiotic *P*, while function $\phi _{P,QR\dots }$ denotes the rate at which bacteria that are already resistant to antibiotics *Q*, *R*, ...acquire resistance to antibiotic *P*.

By introducing the state variable *x* that stacks all bacterial populations, the general model in the presence of *m* antibiotics with concentration $u = [u_1\,\, \dots \,\, u_m]^\top$ can be written compactly as

16 $$\begin{aligned} \dot{x}(t) = \psi ({\textbf {1}}^\top x(t)) x(t) + \Big [ \sum _{j=1}^m F_j(x(t)) u_j(t) \Big ]x(t), \end{aligned}$$

by suitably constructing the matrices $F_j(x)$.

#### Example 3.1

In the case of $m=3$ antibiotics,

$$\begin{aligned} x = [s~~r_A~~r_B~~r_C~~r_{AB}~~r_{AC}~~r_{BC}~~r_{ABC}]^\top \end{aligned}$$

and the corresponding matrices are

$$\begin{aligned} F_1(x) = \begin{bmatrix} -(\mu _A+\phi _A) &  0 &  0 &  0 &  0 &  0 &  0 &  0\\ \phi _A &  0 &  0 &  0 &  0 &  0 &  0 &  0\\ 0 &  0 &  -(\mu _{A,B}+\phi _{A,B}) &  0 &  0 &  0 &  0 &  0\\ 0 &  0 &  0 &  -(\mu _{A,C}+\phi _{A,C}) &  0 &  0 &  0 &  0\\ 0 &  0 &  \phi _{A,B} &  0 &  0 &  0 &  0 &  0\\ 0 &  0 &  0 &  \phi _{A,C} &  0 &  0 &  0 &  0\\ 0 &  0 &  0 &  0 &  0 &  0 &  -(\mu _{A,BC}+\phi _{A,BC}) &  0\\ 0 &  0 &  0 &  0 &  0 &  0 &  \phi _{A,BC} &  0 \end{bmatrix}, \end{aligned}$$

$$\begin{aligned} F_2(x) = \begin{bmatrix} -(\mu _B+\phi _B) &  0 &  0 &  0 &  0 &  0 &  0 &  0\\ 0 &  -(\mu _{B,A}+\phi _{B,A}) &  0 &  0 &  0 &  0 &  0 &  0\\ \phi _B &  0 &  0 &  0 &  0 &  0 &  0 &  0\\ 0 &  0 &  0 &  -(\mu _{B,C}+\phi _{B,C}) &  0 &  0 &  0 &  0\\ 0 &  \phi _{B,A} &  0 &  0 &  0 &  0 &  0 &  0\\ 0 &  0 &  0 &  0 &  0 &  -(\mu _{B,AC}+\phi _{B,AC}) &  0 &  0\\ 0 &  0 &  0 &  \phi _{B,C} &  0 &  0 &  0 &  0\\ 0 &  0 &  0 &  0 &  0 &  \phi _{B,AC} &  0 &  0 \end{bmatrix}, \end{aligned}$$

$$\begin{aligned} F_3(x) = \begin{bmatrix} -(\mu _C+\phi _C) &  0 &  0 &  0 &  0 &  0 &  0 &  0\\ 0 &  -(\mu _{C,A}+\phi _{C,A}) &  0 &  0 &  0 &  0 &  0 &  0\\ 0 &  0 &  -(\mu _{C,B}+\phi _{C,B}) &  0 &  0 &  0 &  0 &  0\\ \phi _C &  0 &  0 &  0 &  0 &  0 &  0 &  0\\ 0 &  0 &  0 &  0 &  -(\mu _{C,AB}+\phi _{C,AB}) &  0 &  0 &  0\\ 0 &  \phi _{C,A} &  0 &  0 &  0 &  0 &  0 &  0\\ 0 &  0 &  \phi _{C,B} &  0 &  0 &  0 &  0 &  0\\ 0 &  0 &  0 &  0 &  \phi _{B,AB} &  0 &  0 &  0 \end{bmatrix}. \end{aligned}$$

Also in the most general scenario, we are interested in investigating Problem 2.1, which can be formulated as an optimal control problem and solved using numerical methods.

## Optimal Multi-Drug Therapy: Problem Formulation and Theoretical Guarantees

In this section, we formulate a finite-horizon optimal control problem aimed at minimising a metric that takes into account both the total number of bacteria and the drug concentration, so as to address two conflicting goals: clearing the infection and minimising the use of antibiotics, thus limiting their side effects, including the fostering of antimicrobial resistance. In particular, suitably choosing the weights in the considered cost functional allows one to solve Problem 2.1, namely, find viable drug concentrations such that the system trajectories reach the safe set in finite time, within the prescribed horizon.

We first highlight a property of the rate functions associated with the onset of resistance, which will be important in the following.

### Remark 4.1

Functions $\phi _X \Big (\frac{r_Y(t)}{b(t)}\Big ){:}{=} \eta _X + \nu _X \frac{r_Y(t)}{b(t)}$ are bounded between $\eta _X$ and $\eta _X + \nu _X$, since $\frac{r_Y(t)}{b(t)} \in [0, 1]$ for all bacterial populations.

We first address the optimal control problem for the two-antibiotic case, with $x(t) = [s(t) \,\, r_A(t) \,\, r_B(t) \,\, r_{AB}(t)]^\top$, over a finite horizon $T>0$.

We assume that the total drug concentration is constrained as

17 $$\begin{aligned} 0 \le u_A(t) + u_B(t) \le {\bar{u}} \end{aligned}$$

and hence $u=[u_A\,\,u_B]^\top$ must lie within the closed, convex, and bounded admissible set

18 $$\begin{aligned} {\mathcal {U}} = \{ u \in {\mathcal {L}}^2([0,T]; {\mathbb {R}}^2) :u_A(t), u_B(t) \ge 0, \; u_A(t)+u_B(t) \le {\bar{u}} \text{ a.e. } \}. \end{aligned}$$

We aim at minimising the cost functional

19 $$\begin{aligned} J(u)=J(x(u),u)=\xi _1 \int _0^T \left( {\textbf {1}}^\top x(t) + \lambda ^\top u(t)\right) \text {d}t + \xi _2 {\textbf {1}}^\top x(T), \end{aligned}$$

where $\lambda = [ \lambda _1 \,\, \lambda _2]^{\top }$, $\lambda _i \ge 0$, while $\xi _1 \ge 0$ and $\xi _2 \ge 0$.

The resulting optimal control problem is

20 $$\begin{aligned} \begin{aligned}&\min _u \; \; \xi _1 \int _0^T \left( {\textbf {1}}^\top x(t) + \lambda ^\top u(t)\right) \text {d}t + \xi _2 {\textbf {1}}^\top x(T)\\&\text{ s.t. } \; \;\; \dot{x}(t) = \psi ({\textbf {1}}^\top x(t)) x(t) + [F_A(x(t)) u_A(t) +F_B(x(t)) u_B(t)] x(t) \\&\; \;\; \; \;\; \; \;\; u \in {\mathcal {U}} \end{aligned} \end{aligned}$$

### Remark 4.2

Solving Problem 2.1 is equivalent to setting $\xi _1=0$ and $\xi _2=1$. We consider a more general cost functional so as to be able to take into account other aspects, such as minimising the side effects of the antibiotics (including the facilitation of the onset of resistance), which are assumed to be proportional to the integral of their concentration over the considered horizon.

In the following, we denote with $\Vert \cdot \Vert _2$ the Euclidean norm (namely, $\Vert v \Vert _2 = \sqrt{v_1^2 + v_2^2 + \dots + v_n^2}$ for $v \in {\mathbb {R}}^n$ and $\Vert A \Vert _2 = \sigma _{\max }(A)$, the largest singular value of *A*, for $A \in {\mathbb {R}}^{m \times n}$), and with $\Vert \cdot \Vert _{{\mathcal {L}}^2}$ the ${\mathcal {L}}^2$ norm (namely, $\Vert f \Vert _{{\mathcal {L}}^2} = \sqrt{\int _0^T \Vert f(t)\Vert _2^2\text {d}t}$ for $f \in {\mathcal {L}}^2([0,T];{\mathbb {R}}^n)$).

For any fixed $u=[u_A\,\,u_B]^\top \in {\mathcal {U}}$, consider the function corresponding to the right-hand-side of system (12):

$$\begin{aligned} f(x,t){:}{=} \left[ \psi ({\textbf {1}}^\top x) + F_A(x) u_A(t) + F_B(x) u_B(t)\right] x, \end{aligned}$$

with $\psi ({\textbf {1}}^\top x)=\alpha -\frac{\beta }{1+\gamma {\textbf {1}}^\top x}$. The function *f*(*x*, *t*) is continuous in *x* for almost all *t*, and measurable in *t* for each *x*. Also, *f* is Lipschitz-continuous in *x*. In fact, for any $x_1,x_2 \in {\mathbb {R}}^4$,

$$\begin{aligned} \Vert f(x_1,t) -f(x_2,t)\Vert _2 \le L \Vert x_1-x_2\Vert _2, \end{aligned}$$

where $L{:}{=} \alpha + \beta + \Vert \bar{F}_A\Vert _2 u_A + \Vert \bar{F}_B\Vert _2 u_B$, where $\bar{F}_A$ and $\bar{F}_B$ correspond to the matrices $F_A$ and $F_B$ where all functions $\phi _X$ have been replaced by a constant value in $[\eta _X, \eta _X + \nu _X]$, in light of Remark 4.1, so as to maximise the 2-norm, and $0 < \frac{\beta }{1+\gamma b(t)} \le \beta$. Moreover, for a given $x \in {\mathbb {R}}^4$, $\Vert f(x,t)\Vert _2 \le L \Vert x\Vert _2$, $t \in [0,T]$. Therefore, given any *T*, Carathéodory’s theorem (see e.g. [54, Theorem II.3.2]) ensures the existence and uniqueness of an absolutely continuous solution, $x \in {\mathcal {C}}([0,T])$ with $\dot{x} \in {\mathcal {L}}^1([0,T])$ a.e. in [0, *T*], for any initial condition $x(0)=x_0$. Also, since *f*(*x*, *t*) is the sum of a continuous function and of products between continuous functions and $u_A,u_B \in {\mathcal {L}}^2([0,T])$, it is $\dot{x} \in {\mathcal {L}}^2([0,T])$ and thus $x \in {\mathcal {H}}^1([0,T])$. As a consequence, the control-to-state map $u \mapsto x(u) \in {\mathcal {H}}^1([0,T])$ is well-posed.

Now we can prove the following estimates on *x*(*t*) and $\dot{x}(t)$.

### Proposition 4.1

For any $u=[u_A\,\,u_B]^\top \in {\mathcal {U}}$, the solution *x*(*t*) of the dynamical system (12) emanating from the initial condition $x_0$ in [0, *T*] satisfies the uniform bound

21 $$\begin{aligned} \Vert x \Vert _{{\mathcal {L}}^2} + \Vert \dot{x} \Vert _{{\mathcal {L}}^2} \le \varphi (T, x_0, {\bar{u}}), \end{aligned}$$

for some $$\varphi $$.

### Proof

Since the solution $x :[0,T] \rightarrow {\mathbb {R}}^n$ emanating from $x(0)=x_0$ has the form

$$\begin{aligned} x(t) = x_0 + \int _0^t \left( \alpha - \frac{\beta }{1+ \gamma b(s)} + F_A u_A(s) + F_B u_B(s) \right) x(s) \,\text {d}s, \end{aligned}$$

for any $t \in [0,T]$, it holds

$$\begin{aligned} \Vert x(t)\Vert _2&\le \Vert x_0\Vert _2 + \int _0^t \left( \alpha + \beta + \Vert \bar{F}_A\Vert _2 u_A(s) + \Vert \bar{F}_B\Vert _2 u_B(s) \right) \Vert x(s)\Vert _2 \,\text {d}s\\&\le \zeta _1 + \int _0^t \zeta _2 \Vert x(s)\Vert _2 \, \text {d}s, \end{aligned}$$

where $\zeta _1 = \Vert x_0\Vert _2$ and $\zeta _2 = \alpha + \beta + \Vert \bar{F}_A\Vert _2 {\bar{u}} + \Vert \bar{F}_B\Vert _2 {\bar{u}}$ since $u_A + u_B \le {\bar{u}}$.

Applying Grönwall’s inequality yields

$$\begin{aligned} \Vert x(t)\Vert _2 \le \zeta _1 \exp \left( \int _0^t \zeta _2\text {d}s \right) \le \zeta _1 \exp \left( \zeta _2 T \right) . \end{aligned}$$

Then,

$$\begin{aligned} \Vert x \Vert _{{\mathcal {L}}^2} \le \sqrt{T} \, \zeta _1 \exp \left( \zeta _2 T \right) {:}{=} \varphi _1(T,x_0, {\bar{u}}). \end{aligned}$$

Moreover, a.e. $$\Vert \dot{x}(t)\Vert _2 \le \zeta _2 \Vert x(t)\Vert _2 \le \zeta _2 \zeta _1 \exp \left( \zeta _2 T \right) $$. Hence,

$$\begin{aligned} \Vert \dot{x}\Vert _{{\mathcal {L}}^2} \le \sqrt{T} \zeta _2 \zeta _1 \exp \left( \zeta _2 T \right) {:}{=} \varphi _2(T,x_0, {\bar{u}}). \end{aligned}$$

The bound (21) follows with $\varphi (T,x_0, {\bar{u}}){:}{=} \varphi _1(T,x_0, {\bar{u}})+\varphi _2(T,x_0, {\bar{u}})$. $\square$

With this preparation, we can now prove the existence of a global optimal control for our problem. The result follows from classical arguments for the existence of solutions in the theory of optimal control problems [14, 20]; we provide a detailed proof for completeness.

### Theorem 4.1

The optimal control problem (20) has a solution $u^* = [u_A^* \,\, u_B^*]^\top$ in ${\mathcal {U}}$ associated with the optimal trajectory $x^*\in {\mathcal {H}}^1([0,T])$.

### Proof

The cost functional *J* is bounded from below (because it is nonnegative), and hence there exists a minimising sequence $\{(x_k, u_{k})\} \subset {\mathcal {H}}^1 \times {\mathcal {U}}$, where $x_k = x(u_{k})$, such that $\lim _{k \rightarrow \infty } J(x_k, u_{k}) = \inf _{v \in {\mathcal {U}}} J(x(v), v) \ge 0$.

Since ${\mathcal {U}}$ is a bounded set in ${\mathcal {L}}^2([0,T])$, it is weakly sequentially compact. Then, from any minimising sequence we can extract a weakly converging subsequence $\{u_{k_m}\}$ with $u_{k_m} \rightharpoonup u^*$. By the Banach-Saks theorem [46], since ${\mathcal {U}}$ is closed and convex, $u^* \in {\mathcal {U}}$. By Proposition 4.1, the corresponding $\{x_{k_m}\}$, with $x_{k_m}= x(u_{k_m})$, is bounded in the Hilbert space ${\mathcal {H}}^1([0,T])$. The compact embedding ${\mathcal {H}}^1([0,T]) \subset \subset {\mathcal {C}}([0,T])$ [1] ensures that there exists a converging subsequence $\{x_{{k_m}_j}\}$ such that $x_{{k_m}_j} \rightarrow x^*$ in ${\mathcal {C}}([0,T])$. Clearly $u_{{k_m}_j} \rightharpoonup u^*$ and, furthermore, $x^* = x(u^*)$ in view of [57, Theorem 1, Section 2.8]. Hence, denoting ${k_m}_j$ with *k* for simplicity, it holds

$$\begin{aligned} J(x^*, u^*)= &   \xi _1 \int _0^T \left( {\textbf {1}}^\top x^*(t) + \lambda ^\top u^*(t)\right) \text {d}t + \xi _2 {\textbf {1}}^\top x^*(T) \\= &   \lim _{k\rightarrow \infty } \xi _1 \int _0^T \left( {\textbf {1}}^\top x_k(t) + \lambda ^\top u_{k}(t)\right) \text {d}t + \xi _2 {\textbf {1}}^\top x_n(T)\\= &   \lim _{k\rightarrow \infty } J(x_k, u_k) = \inf _{v \in {\mathcal {U}}} J(x(v), v). \end{aligned}$$

Thus, $(x^*, u^*)$ is an optimal solution to our control problem. $\square$

All the results stated and proven above can be generalised to the case of *m* antibiotics having concentration $u = [u_1 \,\, \dots \,\, u_m]^\top$, when the optimal control problem can be formulated as

22 $$\begin{aligned} \begin{aligned}&\min _u \; \; \xi _1 \int _0^T \left( {\textbf {1}}^\top x(t) + \lambda ^\top u(t)\right) \text {d}t + \xi _2 {\textbf {1}}^\top x(T),\\&\; \;\;\; \;\;\lambda ^\top = [\lambda _1 \dots \lambda _m],\; \lambda _i \ge 0 \\&\text{ s.t. } \; \;\; \dot{x}(t) = \psi ({\textbf {1}}^\top x(t)) x(t) + \Big [ \sum _{j=1}^m F_j(x) u_j(t) \Big ]x(t) \\&u \in \Big \{v \in {\mathcal {L}}^2([0,T];{\mathbb {R}}^m) :v_j(t) \ge 0, \;\;\; \sum _{j=1}^m v_j(t) \le {\bar{u}} \;\; \text{ a.e. } \text{ in } [0,T]\Big \}. \end{aligned} \end{aligned}$$

The existence of an optimal control that solves the above problem can be proved through the same arguments used in the case of two antibiotics.

### Necessary Conditions for Optimality

Without loss of generality, we consider the case of two antibiotics and derive the optimality system by using the Pontryagin minimum principle. To this aim, we consider the Hamiltonian function, defined as

$$\begin{aligned} H(x, u, p)&{:}{=} \xi _1({\textbf {1}}^\top x + \lambda ^\top u) + p^\top [\psi ({\textbf {1}}^\top x)x + F_A(x)u_A x + F_B(x)u_B x] \\&= \xi _1 {\textbf {1}}^\top x + \xi _1 \lambda ^\top u + p^\top \psi ({\textbf {1}}^\top x)x + p^\top F_A(x)u_A x + p^\top F_B(x)u_B x, \end{aligned}$$

where $p(t) = [p_s(t) \,\, p_{r_A}(t) \,\, p_{r_B}(t) \,\, p_{r_{AB}}(t)]^\top$ is the costate (adjoint) vector.

The state equation can be derived from the Hamiltonian as

$$\begin{aligned} \dot{x}(t) = \nabla _p H = \psi ({\textbf {1}}^\top x)x + F_A(x)u_A x + F_B(x)u_B x, \end{aligned}$$

with initial condition $x(0) = x_0$, while the costate equation is given by

$$\begin{aligned} \dot{p}(t) = -\nabla _x H. \end{aligned}$$

To compute it, we need to evaluate

$$\begin{aligned} \nabla _x H = \xi _1 {\textbf {1}} + \nabla _x[p^\top \psi ({\textbf {1}}^\top x)x] + \nabla _x[p^\top F_A(x)u_A x] + \nabla _x[p^\top F_B(x)u_B x]. \end{aligned}$$

We have $\nabla _x[p^\top \psi ({\textbf {1}}^\top x)x] = \psi '({\textbf {1}}^\top x)(p^\top x){\textbf {1}} + \psi ({\textbf {1}}^\top x)p$ for the term involving $\psi$, while $\nabla _x[p^\top F_A(x)u_A x] = u_A[\nabla _x(F_A(x)x)]^\top p = u_A[F_A(x) + (\nabla _x F_A(x))x]^\top p$ and, similarly, $\nabla _x[p^\top F_B(x)u_B x] = u_B[F_B(x) + (\nabla _x F_B(x))x]^\top p$. Therefore, the costate equation becomes

23 $$\begin{aligned} \begin{array}{ll} \dot{p}(t) = & -\xi _1 {\textbf {1}} - \psi '({\textbf {1}}^\top x)(p^\top x){\textbf {1}} - \psi ({\textbf {1}}^\top x)p \\ & - u_A[F_A(x) + (\nabla _x F_A(x))x]^\top p - u_B[F_B(x) + (\nabla _x F_B(x))x]^\top p, \end{array} \end{aligned}$$

with transversality condition $p(T) = \xi _2 {\textbf {1}}$.

The gradient of the Hamiltonian with respect to the control is

$$\begin{aligned} \nabla _u H = \xi _1 \lambda + \begin{bmatrix} p^\top F_A(x)x \\ p^\top F_B(x)x \end{bmatrix}. \end{aligned}$$

The optimal control satisfies $u^*(t) = \arg \min _{u \in {\mathcal {U}}} H(x(t), u, p(t))$, which is equivalent to $u^*(t) = \arg \min _{u \in {\mathcal {U}}} \{\xi _1 \lambda ^\top u + p^\top [F_A(x)u_A x + F_B(x)u_B x]\}$ or, more explicitly,

$$\begin{aligned} u^*(t) = \arg \min _{\begin{array}{c} u_A, u_B \ge 0 \\ u_A + u_B \le \bar{u} \end{array}} \{\xi _1 \lambda _1 u_A + \xi _1 \lambda _2 u_B + u_A p^\top F_A(x)x + u_B p^\top F_B(x)x\}. \end{aligned}$$

Then, defining the switching functions

24 $$\begin{aligned} \sigma _A(t)&{:}{=} \xi _1 \lambda _1 + p(t)^\top F_A(x(t))x(t), \end{aligned}$$

25 $$\begin{aligned} \sigma _B(t)&{:}{=} \xi _1 \lambda _2 + p(t)^\top F_B(x(t))x(t), \end{aligned}$$

the optimal control problem reduces to

$$\begin{aligned} u^*(t) = \arg \min _{\begin{array}{c} u_A, u_B \ge 0 \\ u_A + u_B \le \bar{u} \end{array}} \{\sigma _A(t) u_A + \sigma _B(t) u_B\}. \end{aligned}$$

The optimality system for the two-antibiotic optimal control problem, consisting of coupled systems of differential equations, is summarised next.

#### Theorem 4.2

Let $(x^*, u^*)$ be an optimal state-control pair for the optimal control problem (20). Then, there exists a costate function $p :[0,T] \rightarrow {\mathbb {R}}^4$ such that the following conditions hold:

1. **State equation (forward in time):**
  $$\begin{aligned} \dot{x}^*(t) = \psi ({\textbf {1}}^\top x^*)x^* + F_A(x^*)u_A^* x^* + F_B(x^*)u_B^* x^*, \quad x^*(0) = x_0; \end{aligned}$$
2. **Costate equation (backward in time):**
  $$\begin{aligned} \dot{p}(t) =&-\xi _1 {\textbf {1}} - \psi '({\textbf {1}}^\top x)(p^\top x){\textbf {1}} - \psi ({\textbf {1}}^\top x)p \\&- u_A[F_A(x) + (\nabla _x F_A(x))x]^\top p - u_B[F_B(x) + (\nabla _x F_B(x))x]^\top p, \\ p(T) =&\xi _2 {\textbf {1}}; \end{aligned}$$
3. **Optimality condition:**
  $$\begin{aligned} u^*(t) = \arg \min _{u \in {\mathcal {U}}} \{\sigma _A(t) u_A + \sigma _B(t) u_B\}, \end{aligned}$$
  with ${\mathcal {U}} = \{ u \in {\mathcal {L}}^2([0,T]; {\mathbb {R}}^2) :u_A(t), u_B(t) \ge 0, \; u_A(t)+u_B(t) \le {\bar{u}} \text{ a.e. } \}$ and switching functions
  $$\begin{aligned} \sigma _A(t)&= \xi _1 \lambda _1 + p(t)^\top F_A(x^*(t))x^*(t), \\ \sigma _B(t)&= \xi _1 \lambda _2 + p(t)^\top F_B(x^*(t))x^*(t). \end{aligned}$$

The optimality system forms a two-point boundary value problem (TPBVP) that can be solved numerically using methods such as shooting methods, finite difference methods, or direct transcription methods.

Analytically, the optimal control can be characterised as follows.

If $\sigma _A(t) < \sigma _B(t)$ and $\sigma _A(t) < 0$, then $u_A^*(t) = \bar{u}$ and $u_B^*(t) = 0$ (only antibiotic *A* is used, with maximum concentration).

If $\sigma _B(t) < \sigma _A(t)$ and $\sigma _B(t) < 0$, then $u_A^*(t) = 0$ and $u_B^*(t) = \bar{u}$ (only antibiotic *B* is used, with maximum concentration).

If $\sigma _A(t) > 0$ and $\sigma _B(t) > 0$, then $u_A^*(t) = 0$ and $u_B^*(t) = 0$ (no treatment).

If $\sigma _A(t) = \sigma _B(t) < 0$, then $u_A^*(t) + u_B^*(t) = \bar{u}$ and $u_A^*, u_B^* \ge 0$ (any combination with total concentration $\bar{u}$ can be equivalently used).

If in some interval $[t_1,t_2]$, with $t_1 < t_2$, it is either $\sigma _A(t) = \sigma _B(t) = 0$, or $\sigma _A(t) = 0$ and $\sigma _B(t) > 0$, or $\sigma _A(t) > 0$ and $\sigma _B(t) = 0$, then the system exhibits singular arcs. In this case, the standard optimality condition becomes indeterminate and, as discussed in the following subsection, higher-order conditions must be employed to determine singular controls; see, e.g., [38, 39].

### Singular Controls

We keep considering the case of two antibiotics, without loss of generality. We derive the singular controls by repeatedly differentiating the vanishing switching functions and using the state and costate equations as expressions for $\dot{x}$ and $\dot{p}$. The details are given in the Appendix. The key structural scalar is the *Lie bracket coefficient*:

$$\begin{aligned} q \equiv p^\top [f_A, f_B](x) = p^\top J_A f_B - p^\top J_B f_A, \end{aligned}$$

where $[f_A, f_B](x) \equiv J_A f_B - J_B f_A$ is the Lie bracket of the control vector fields. The value of *q* on the singular arc governs whether the controls are determined at the first or the second order.

In all the following cases, to be admissible, the computed singular control must satisfy the box constraints: $u_A^{\text {sing}} \ge 0$, $u_B^{\text {sing}} \ge 0$, $u_A^{\text {sing}} + u_B^{\text {sing}} \le {\bar{u}}$.

#### Fully Singular Arc, Case $q \ne 0$: First-Order Singular Control

Consider $\sigma _A(t) = \sigma _B(t) = 0$ (fully singular arc). When the Lie bracket coefficient *q* is nonzero, differentiating $\sigma _A$ and $\sigma _B$ once along the optimal trajectory yields a $2\times 2$ linear system in $(u_A, u_B)$ whose off-diagonal terms are $\pm q$. In fact, the diagonal terms in $({\dot{\sigma }}_A, {\dot{\sigma }}_B)$ that involve $u_A$ and $u_B$, respectively, cancel out due to the structure of the Hamiltonian, and only the off-diagonal coupling through *q* survives. Solving the linear system yields

$$\begin{aligned} u_A^{\text {sing}} = \frac{C_B}{q}, \qquad u_B^{\text {sing}} = -\frac{C_A}{q}, \end{aligned}$$

where the *drift terms* $C_A$, $C_B$ collect all control-independent contributions:

$$\begin{aligned} C_A&\equiv -\xi _1{\textbf {1}}^\top f_A - \psi '(b)(p^\top x)({\textbf {1}}^\top f_A) - \psi (b)(p^\top f_A) + \psi (b)\,p^\top J_A x,\\ C_B&\equiv -\xi _1{\textbf {1}}^\top f_B - \psi '(b)(p^\top x)({\textbf {1}}^\top f_B) - \psi (b)(p^\top f_B) + \psi (b)\,p^\top J_B x. \end{aligned}$$

Each optimal concentration is determined by the drift of the *other* switching function.

#### Fully Singular Arc, Case $q = 0$: Second-Order Singular Control

When $\sigma _A(t) = \sigma _B(t) = 0$ (fully singular arc) and $q = 0$, the first-order conditions reduce to $C_A = C_B = 0$, which are consistency conditions on the state-costate pair, but provide no information on the controls. Hence, a second differentiation is required. Setting $\ddot{\sigma }_A = \ddot{\sigma }_B = 0$ yields the linear system

26 $$\begin{aligned} {\tilde{Q}}\,u^{\text {sing}} = -{\tilde{C}}, \qquad {\tilde{Q}} \equiv \begin{pmatrix}{\tilde{Q}}_{AA} &  {\tilde{Q}}_{AB}\\ {\tilde{Q}}_{BA} &  {\tilde{Q}}_{BB}\end{pmatrix}, \qquad {\tilde{C}} \equiv \begin{pmatrix}{\tilde{C}}_A\\ {\tilde{C}}_B\end{pmatrix}, \end{aligned}$$

where the explicit expressions for the entries of ${\tilde{Q}}$ and the drift ${\tilde{C}}$ are given in the Appendix. If $\det ({\tilde{Q}}) \ne 0$, the singular control is uniquely determined by Cramer’s rule:

$$\begin{aligned} u_A^{\text {sing}} = \frac{-{\tilde{Q}}_{BB}{\tilde{C}}_A + {\tilde{Q}}_{AB}{\tilde{C}}_B}{\det ({\tilde{Q}})}, \qquad u_B^{\text {sing}} = \frac{{\tilde{Q}}_{BA}{\tilde{C}}_A - {\tilde{Q}}_{AA}{\tilde{C}}_B}{\det ({\tilde{Q}})}. \end{aligned}$$

The generalised Legendre-Clebsch (GLC) [13, 26] condition for optimality requires that ${\tilde{Q}}$, the coefficient matrix of the controls in $(\ddot{\sigma }_A, \ddot{\sigma }_B)$, is positive semi-definite on the admissible cone. A sufficient condition is:

$$\begin{aligned} {\tilde{Q}}_{AA} \ge 0, \qquad {\tilde{Q}}_{BB} \ge 0, \qquad \det ({\tilde{Q}}) > 0. \end{aligned}$$

#### Mixed Singular Arcs

When exactly one switching function vanishes, while the other is strictly positive, the bang condition immediately pins one control to zero, and only the remaining control is singular.

Consider the case $\sigma _A = 0$ and $\sigma _B > 0$. Since $\sigma _B > 0$, Pontryagin’s minimum principle forces $u_B^* = 0$. Then, differentiating $\sigma _A = 0$ twice along the reduced dynamics yields

$$\begin{aligned} u_A^{\text {sing}} = -\frac{{\tilde{C}}_A^{\,(0)}}{{\tilde{Q}}_{AA}}, \end{aligned}$$

where ${\tilde{C}}_A^{\,(0)}$ is the drift ${\tilde{C}}_A$ evaluated at $u_B = 0$, and ${\tilde{Q}}_{AA}$ is the (1, 1) entry of the matrix in (26). The GLC condition is:

$$\begin{aligned} {\tilde{Q}}_{AA} > 0. \end{aligned}$$

Admissibility requires $0 \le u_A^{\text {sing}} \le {\bar{u}}$, and the assumption $\sigma _B > 0$ must be verified a posteriori along the resulting trajectory. By symmetry, when $\sigma _B = 0$, $\sigma _A > 0$, Pontryagin’s minimum principle forces $u_A^*(t) = 0$ and then, differentiating $\sigma _B = 0$ twice along the reduced dynamics, it follows:

$$\begin{aligned} u_B^{\text {sing}} = -\frac{{\tilde{C}}_B^{\,(0)}}{{\tilde{Q}}_{BB}}, \end{aligned}$$

with GLC condition ${\tilde{Q}}_{BB} > 0$ and consistency check $\sigma _A > 0$ a posteriori.

### An Alternative Model Predictive Control Approach

Implementing the optimal control solution in a clinical setting can be challenging, and critical in the presence of disturbances and model uncertainty. In fact, the open-loop optimal control solution is computed once, offline, under the nominal model dynamics, and then applied without any correction. In practice, however, bacterial dynamics in the host are subject to patient-specific variability and unmodelled phenomena, and hence a controller that does not react to the evolving state of the system may perform poorly or even fail to drive the trajectory into the safe set. For this reason, in realistic settings and in practical cases, it can be worth seeking an alternative solution to Problem 2.1 through a model predictive control (MPC) scheme [45] that retains the cost functional, control constraints and system dynamics constraints of the optimal control problem, while solving it over a receding finite horizon. The receding-horizon implementation yields a closed-loop control law by recomputing the control sequence at each sampling instant based on the current state. This inherent feedback mechanism improves robustness to disturbances, noise and model uncertainty compared to open-loop optimal control.

In particular, we consider a finite-horizon model predictive control strategy, where the antibiotic concentration to be administrated is re-assessed at a regular interval $\tau$, thus leading to piecewise constant drug concentrations; $\tau$ is thus the sampling time to both retrieve the state measurements and change the therapy accordingly. Re-assessing the therapy at regular intervals is in line with clinical practice. The resulting finite-horizon model predictive control scheme incorporates a discrete-time version of system (16), obtained through forward Euler discretisation with the discrete interval $\tau _d$, and hence entails solving, at each instant $t=k\tau$, $k = 0,1,\dots$, the optimisation problem

27a $$\begin{aligned}&\min \; \; J_k \doteq \xi _1\sum _{\kappa \in {\mathcal {T}}_k}\left( {\textbf {1}}^\top x(\kappa ) + \lambda ^\top u(\kappa )\right) +\xi _2{\textbf {1}}^\top x(k+N_{\text {h}}), \end{aligned}$$

27b $$\begin{aligned}&\lambda ^\top = [\lambda _1, \dots , \lambda _m],\; \lambda _i \ge 0 \end{aligned}$$

27c $$\begin{aligned}&\text{ s.t. } \;\forall \,\kappa \in {\mathcal {T}}_k, \nonumber \\&x(\kappa +1) = x(\kappa )+\tau _d\left( \psi ({\textbf {1}}^\top x(\kappa )) x(\kappa ) + \Big [ \sum _{j=1}^m F_j(x(\kappa )) u_j(\kappa ) \Big ]x(\kappa ) \right) \end{aligned}$$

27d $$\begin{aligned}&u_j(\kappa )\ge 0, \quad \sum _{j=1}^m u_j(\kappa ) \le {\bar{u}}, \end{aligned}$$

where $\psi ({\textbf {1}}^\top x(\kappa ))=\left[ \alpha - \frac{\beta }{1+\gamma {\textbf {1}}^\top x(\kappa )}\right]$, while ${\mathcal {T}}_k=\{k,\dots ,k+N_{\text {h}}-1\}$ is the prediction horizon and $N_{\text {h}}$ is the horizon length.

#### Remark 4.3

Robustness in MPC design typically involves the explicit specification of uncertainty sets that contain all model mismatches or noise signals compatible with performance, stability, and constraint satisfaction guarantees, and then the adoption of robust MPC schemes such as min-max [41] or constraint tightening [42]. We do not consider explicit state constraints, specific disturbances, or noise, hence such approaches go beyond the scope of our work, but represent an interesting direction for future developments involving parametric uncertainty or disturbances affecting the model.

Finite-time convergence to the safe set immediately follows if asymptotic stability of the equilibrium point at the origin (${\bar{x}} =0$) is guaranteed, since the safe set contains ${\bar{x}} =0$ (which is not on the boundary $b={\bar{b}}$). To guarantee that the finite horizon optimal control problem is feasible – where feasibility stands for both recursive feasibility and stability of the MPC closed-loop – a terminal cost, terminal constraint or invariant terminal set is often included in the optimal control problem; see, e.g., [44]. However, the definition of such additional stabilising ingredients is generally challenging, especially in nonlinear settings. Still, [11, Section 3] shows that considering sufficiently large optimisation horizons ensures closed-loop stability also in the absence of stabilising terminal constraints or costs. Our formulation does not have state constraints, but only input constraints, and hence represents a particular case of the class addressed in [11]. Our specific model and MPC formulation satisfy the assumptions in [11, Section 3]: (I) an equilibrium pair $({\bar{x}}, {\bar{u}})$ exists, for some admissible ${\bar{u}}$, such that the system is controllable to ${\bar{x}}$ locally around the equilibrium: in our case, this is true with ${\bar{x}}=0$ and ${\bar{u}}=0$ (see Proposition 2.2, valid also in the more general multi-antibiotic setting), and the local region is the domain of attraction of the origin for the dynamics; (II) the running cost $\ell (x,u)$ is such that $\ell ({\bar{x}}, {\bar{u}})=0$: this is true in our case, since $\ell (x,u)= {\textbf {1}}^\top x + \lambda ^\top u$. Therefore, when selecting a sufficiently large prediction horizon such that condition (10) in [11, Theorem 4] is satisfied, recursive feasibility and asymptotic stability of the MPC closed-loop are guaranteed.

The MPC solution converges to the solution of the optimal control problem if the length of the prediction horizon converges to infinity; although an infinite prediction horizon cannot be implemented in practice, choosing a large enough horizon allows us to achieve a good enough approximation of the solution to the optimal control problem.

## Numerical Simulations

First, we consider the optimal control problem (22) formulated and analysed in Section 4, aimed at minimising a cost that accounts for both the size of the total bacterial population and the drug concentration over a finite horizon, and we solve it numerically in a realistic setting, with the system parameters chosen following [16]. Then, in the same realistic setting, we consider the alternative approach discussed in Section 4.3 to find a solution to Problem 2.1 in practical applications through a model predictive control scheme [45].

### Optimal Control Solution

We numerically solve the optimal control problem (22) in Section 4 with a multiple shooting method, using CasAdi [5] through its MATLAB interface. This open-loop formulation provides a theoretically grounded benchmark: it establishes the best achievable performance under the considered model, yielding an optimal trade-off between bacterial load and antibiotic usage that serves as a reference for any subsequent control design.

For our numerical simulation campaign, we consider two different case studies, one with $m=2$ antibiotics and the other with $m=3$ antibiotics, hereafter denoted as *A*, *B*, and (when relevant) *C*. The values of the model parameters are chosen as reported in Table 1, following realistic case studies in the biological literature [16]. The value of ${\bar{b}}$ defined in (3), computed according to the parameter values in Table 1, is $\bar{b}= 3.6997 \times 10^{6}$. We also set $\bar{u}=1.5\tfrac{\alpha }{\min _{j\in \{A,B,C\}}{\mu _P}} \approx 2.1889$ as the maximum tolerated drug concentration.

The therapy duration is $T={72}$h and the considered initial conditions, corresponding to different scenarios, are listed in Table 2. The weights of the cost functional are $\xi _1 = \xi _2 = 1$ and $\lambda _i = 0.1$, for $i=1,2,3.$ We denote with $J_T$ the value of the cost functional corresponding to the optimal solution.

**Fig. 5:**
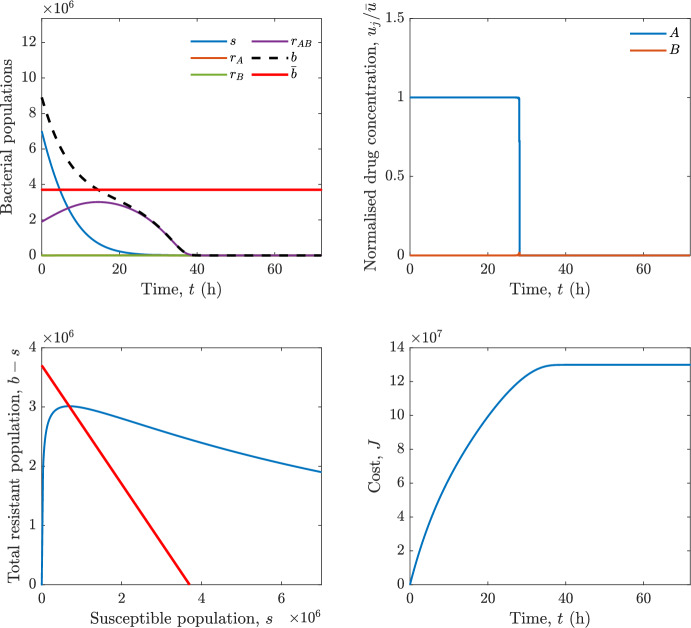
(OCP) Time evolution of the various bacterial populations, with the value of ${\bar{b}}$ marked by the red horizontal line (top left), system trajectory in the plane $b-s$ vs. *s*, where *s* are bacteria that are susceptible to all drugs while $b-s$ denotes the total population of bacteria that are resistant to at least one drug, and the red line delimits the safe set (bottom left), time evolution of the optimal normalised drug concentrations $u_j/{\bar{u}}$ (top right), time evolution of the cost functional *J* (bottom right) in the case with $m=2$ antibiotics, with initial conditions chosen as in scenario 1 in Table 2. The parameters are chosen as described in the main text. The final cost is $J_T={1.29\times 10^8}$.

We begin by considering the case of $m=2$ antibiotics. In scenario 1 in Table 2, only $7\times 10^{6}$ susceptible bacteria and $1.9\times 10^{6}$ bacteria that are resistant to both antibiotics are initially present. The results are shown in Figure 5. As expected, antibiotic *A*, which has the highest killing rate, is chosen and is administered for 28 hours.

Since the optimal control problem aims not only at steering the trajectories into the safe set, but also at minimising the final value of the state as well as the integral of the state, in all considered scenarios antibiotics are administered also after the trajectory has reached the safe set.

**Fig. 6:**
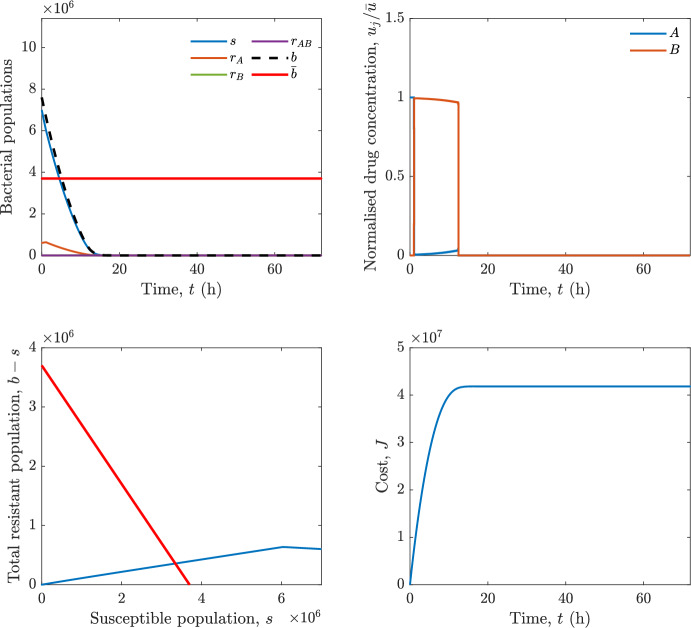
(OCP) Time evolution of the various bacterial populations, with the value of ${\bar{b}}$ marked by the red horizontal line (top left), system trajectory in the plane $b-s$ vs. *s*, where *s* are bacteria that are susceptible to all drugs while $b-s$ denotes the total population of bacteria that are resistant to at least one drug, and the red line delimits the safe set (bottom left), time evolution of the optimal normalised drug concentrations $u_j/{\bar{u}}$ (top right), time evolution of the cost functional *J* (bottom right) in the case with $m=2$ antibiotics, with initial conditions chosen as in scenario 2 in Table 2. The parameters are chosen as described in the main text. The final cost is $J_T={4.18\times 10^7}$.

Next, we consider an initial condition with a reduced amount of bacteria that are resistant to both antibiotics ($1\times 10^{3}$) and the additional significant presence of $6\times 10^{5}$ bacteria that are resistant to antibiotic *A*, in the absence of bacteria that are resistant to antibiotic *B* (scenario 2 in Table 2). This initial configuration leads to the administration of antibiotic *A*, for 1 hour, followed by a combination of both antibiotics, with a prevalence of *B*, for additional 11 hours (Figure 6). The system exhibits singular arcs.

**Fig. 7:**
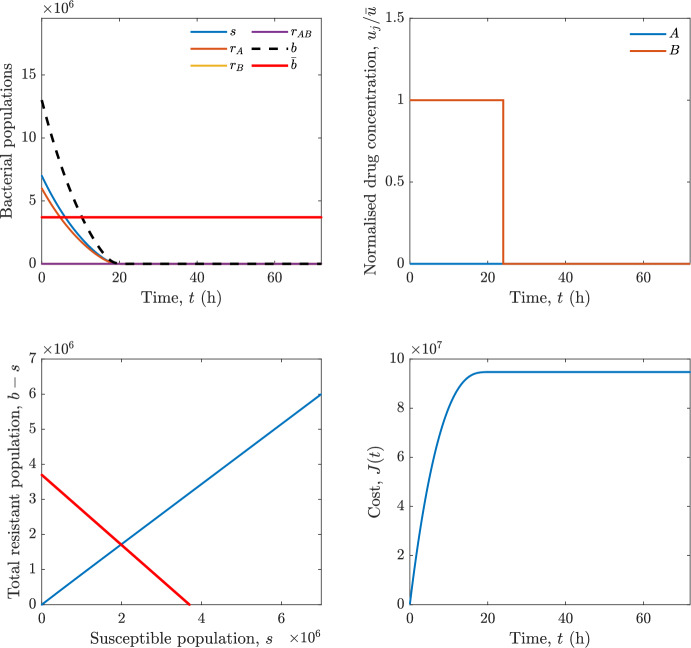
(OCP) Time evolution of the various bacterial populations, with the value of ${\bar{b}}$ marked by the red horizontal line (top left), system trajectory in the plane $b-s$ vs. *s*, where *s* are bacteria that are susceptible to all drugs while $b-s$ denotes the total population of bacteria that are resistant to at least one drug, and the red line delimits the safe set (bottom left), time evolution of the optimal normalised drug concentrations $u_j/{\bar{u}}$ (top right), time evolution of the cost functional *J* (bottom right) in the case with $m=2$ antibiotics, with initial conditions chosen as in scenario 3 in Table 2. The parameters are chosen as described in the main text. The final cost is $J_T={9.47\times 10^7}$.

Further increasing the initial number of bacteria that are resistant to antibiotic *A* to $6\times 10^{6}$, *ceteris paribus* (scenario 3 in Table 2), leads to the administration of antibiotic *B* only, for 23.98 hours (Figure 7).

**Fig. 8:**
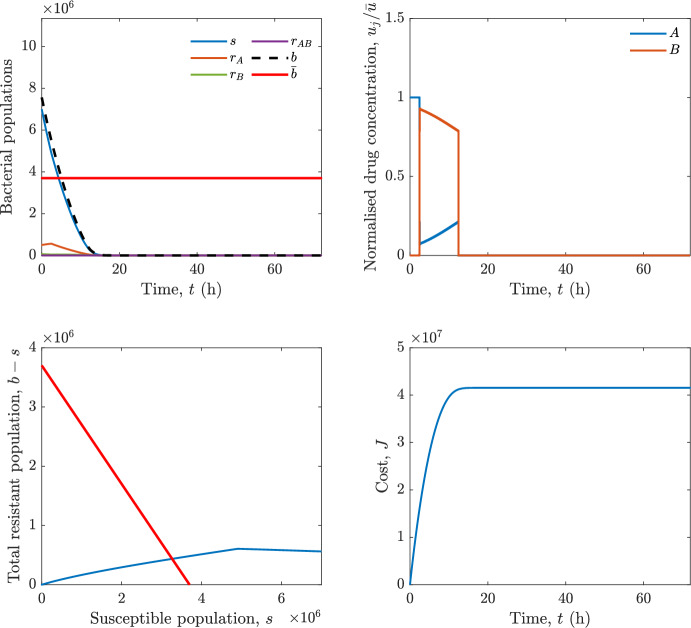
(OCP) Time evolution of the various bacterial populations, with the value of ${\bar{b}}$ marked by the red horizontal line (top left), system trajectory in the plane $b-s$ vs. *s*, where *s* are bacteria that are susceptible to all drugs while $b-s$ denotes the total population of bacteria that are resistant to at least one drug, and the red line delimits the safe set (bottom left), time evolution of the optimal normalised drug concentrations $u_j/{\bar{u}}$ (top right), time evolution of the cost functional *J* (bottom right) in the case with $m=2$ antibiotics, with initial conditions chosen as in scenario 4 in Table 2. The parameters are chosen as described in the main text. The final cost is $J_T={4.15\times 10^7}$.

However, reducing the initial amount of bacteria that are resistant to *A* to $5\times 10^{5}$ and introducing $6\times 10^{4}$ bacteria that are resistant to *B* (scenario 4 in Table 2) leads again to singular controls. Specifically, antibiotic *A* is given for 2 hours, followed by a combination of both with the prevalence of antibiotic *B*, for 10 hours (Figure 8).

**Fig. 9:**
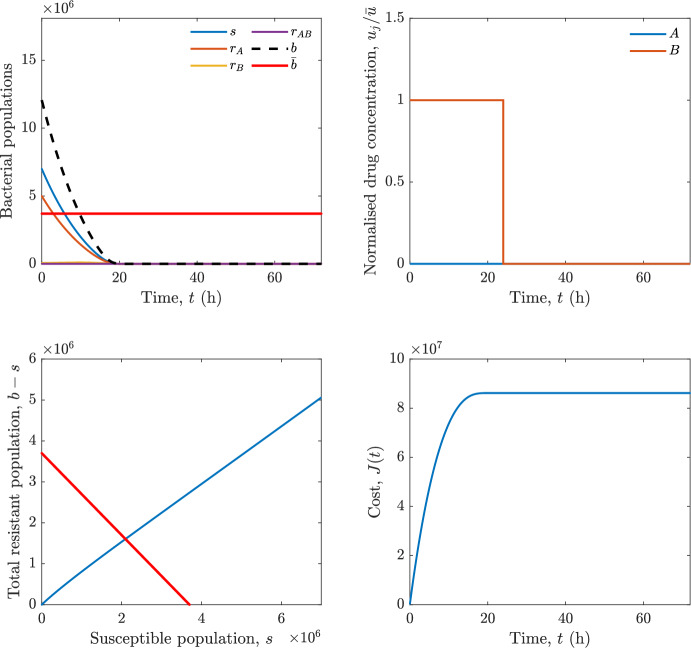
(OCP) Time evolution of the various bacterial populations, with the value of ${\bar{b}}$ marked by the red horizontal line (top left), system trajectory in the plane $b-s$ vs. *s*, where *s* are bacteria that are susceptible to all drugs while $b-s$ denotes the total population of bacteria that are resistant to at least one drug, and the red line delimits the safe set (bottom left), time evolution of the optimal normalised drug concentrations $u_j/{\bar{u}}$ (top right), time evolution of the cost functional *J* (bottom right) in the case with $m=2$ antibiotics, with initial conditions chosen as in scenario 5 in Table 2. The parameters are chosen as described in the main text. The final cost is $J_T={8.61\times 10^7}$.

Increasing the initial amount of bacteria that are resistant to *A* to $5\times 10^{6}$, while keeping $6\times 10^{4}$ bacteria that are resistant to *B* (scenario 5 in Table 2), leads again to the administration of antibiotic *B* for 23.88 hours (Figure 9).

**Fig. 10:**
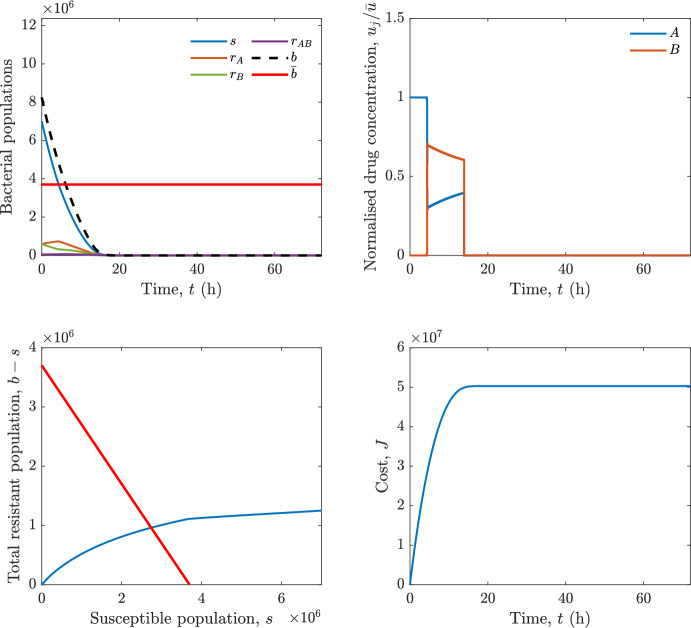
(OCP) Time evolution of the various bacterial populations, with the value of ${\bar{b}}$ marked by the red horizontal line (top left), system trajectory in the plane $b-s$ vs. *s*, where *s* are bacteria that are susceptible to all drugs while $b-s$ denotes the total population of bacteria that are resistant to at least one drug, and the red line delimits the safe set (bottom left), time evolution of the optimal normalised drug concentrations $u_j/{\bar{u}}$ (top right), time evolution of the cost functional *J* (bottom right) in the case with $m=2$ antibiotics, with initial conditions chosen as in scenario 6 in Table 2. The parameters are chosen as described in the main text. The final cost is $J_T={5.029\times 10^7}$.

Finally, in the coexistence scenario 6, the system has singular arcs and the resulting therapy yields the administration of drug *A* for 4 hours, followed by a combination of both drugs, for additional 10 hours; see Figure 10.

**Table 1:** Model parameters (bacterial growth, mutation and horizontal transfer, effect of the immune system, and effect of antibiotics) and their values.

Parameter	Biological meaning	Value	Units
$\alpha$	Bacterial net growth rate	$2.7726^*$	$\hbox {d}^{-1}$
$\beta$	Maximal killing rate of bacteria due to phagocytes	$33.6038 ^*$	$\hbox {d}^{-1}$
$1/\gamma$	Number of activated phagocytes	$332711^*$	-
$\mu _{P}$	Killing rate of bacteria due to drug *P*	${2.1; 1.9; 2}^*$	$\hbox {d}^{-1}$
$\mu _{P,Q}$	Killing rate of bacteria due to drug *P*	2.1; 1.9; 2	$\hbox {d}^{-1}$
$\mu _{P,QR}$	Killing rate of bacteria due to drug *P*	2.1; 1.9; 2	$\hbox {d}^{-1}$
$\nu$	Rate of horizontal transfer	$0.001^*$	$\hbox {d}^{-1}$
$\eta$	Rate of mutations	0.002	$\hbox {d}^{-1}$

Data taken from [16] are marked with *. The killing rate due to antibiotic *P* is: $\mu _P$ for susceptible bacteria, $\mu _{P,Q}$ for bacteria that are resistant to antibiotic *Q*, $\mu _{P,QR}$ for bacteria that are resistant to antibiotics *Q* and *R*, where $P,Q,R\in \{A,B,C\}$. For $\mu _{P,Q}$ and $\mu _{P,QR}$ we set values comparable to those in [16] for $\mu _P$, while $\eta$ has a value of the same order of magnitude as the value of $\nu$ in [16]. In all our simulations, time is expressed in hours, and hence all the parameters in the table with units $\hbox {d}^{-1}$ are divided by 24, so as to convert their unit into $\hbox {h}^{-1}$

**Table 2:** Considered initial conditions for the system dynamics, for the case of $m=2$ and of $m=3$ antibiotics.

Scenario								
($m=2$)	*s*	$r_A$	$r_B$	$r_{AB}$				
1	$7\times 10^{6}$	0	0	$1.9\times 10^{6}$				
2	$7\times 10^{6}$	$6\times 10^{5}$	0	$1\times 10^{3}$				
3	$7\times 10^{6}$	$6\times 10^{6}$	0	$1\times 10^{3}$				
4	$7\times 10^{6}$	$5\times 10^{5}$	$6\times 10^{4}$	$1\times 10^{3}$				
5	$7\times 10^{6}$	$5\times 10^{6}$	$6\times 10^{4}$	$1\times 10^{3}$				
6	$7\times 10^{6}$	$6\times 10^{5}$	$6\times 10^{5}$	$5\times 10^{4}$				

Scenario								
($m=3$)	*s*	$r_A$	$r_B$	$r_C$	$r_{AB}$	$r_{AC}$	$r_{BC}$	$r_{ABC}$
1	$7\times 10^{6}$	0	0	0	0	0	0	$1.9\times 10^{6}$
2	$7\times 10^{6}$	$2\times 10^{4}$	$3\times 10^{5}$	$2\times 10^{2}$	$2\times 10^{2}$	$5\times 10^{1}$	$3\times 10^{1}$	$1\times 10^{3}$
3	$7\times 10^{6}$	$5\times 10^{5}$	$6\times 10^{4}$	$1\times 10^{3}$	$2\times 10^{3}$	$5\times 10^{2}$	$3\times 10^{2}$	$1\times 10^{3}$
4	$7\times 10^{6}$	$6\times 10^{6}$	0	$5\times 10^{5}$	$3\times 10^{1}$	$1\times 10^{5}$	$2\times 10^{2}$	$5\times 10^{4}$

**Fig. 11:**
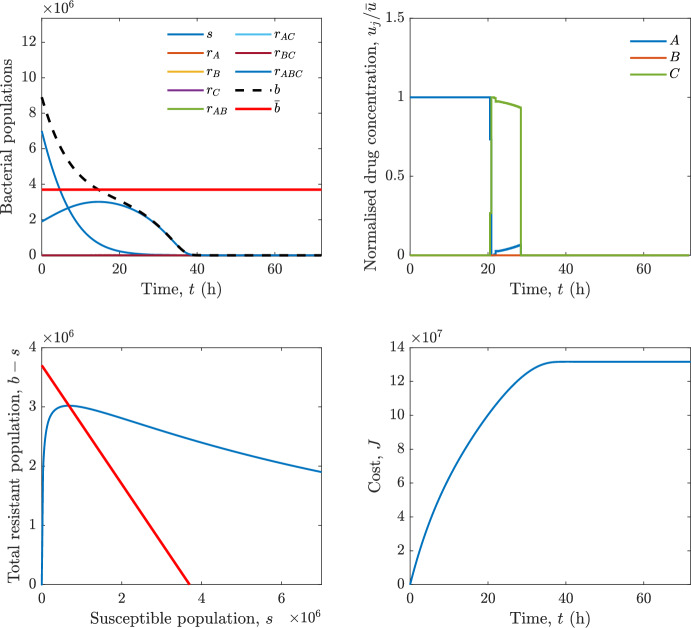
(OCP) Time evolution of the various bacterial populations, with the value of ${\bar{b}}$ marked by the red horizontal line (top left), system trajectory in the plane $b-s$ vs. *s*, where *s* are bacteria that are susceptible to all drugs while $b-s$ denotes the total population of bacteria that are resistant to at least one drug, and the red line delimits the safe set (bottom left), time evolution of the optimal normalised drug concentrations $u_j/{\bar{u}}$ (top right), time evolution of the cost functional *J* (bottom right) in the case with $m=3$ antibiotics, with initial conditions chosen as in scenario 1 in Table 2. The parameters are chosen as described in the main text. The final cost is $J_T={1.30\times 10^8}$.

In the case of $m=3$ antibiotics, when only $7\times 10^{6}$ susceptible bacteria and $1.9\times 10^{6}$ bacteria that are resistant to all antibiotics are initially present (scenario 1 in Table 2), singular controls arise. Indeed, antibiotic *A* is administered for 20 hours, which is again expected since it is the most effective, then it is given in combination with antibiotic *C* for additional 8 hours; see Figure 11.

**Fig. 12:**
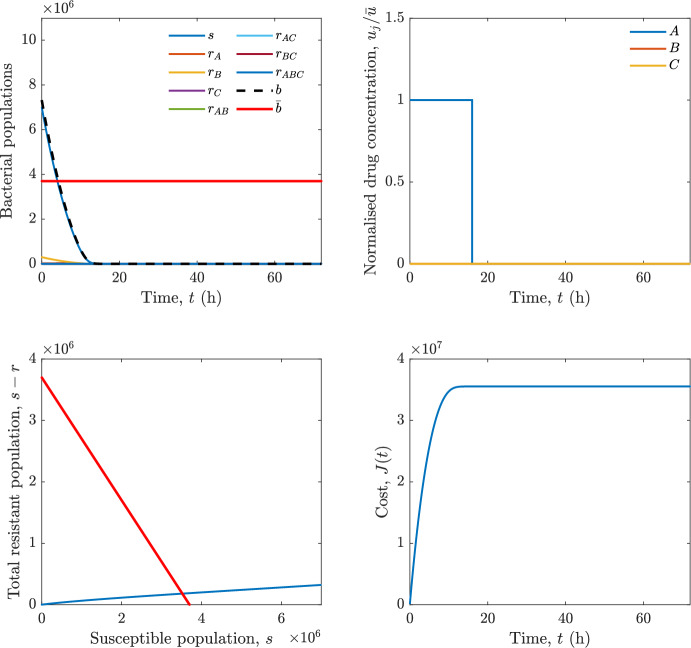
(OCP) Time evolution of the various bacterial populations, with the value of ${\bar{b}}$ marked by the red horizontal line (top left), system trajectory in the plane $b-s$ vs. *s*, where *s* are bacteria that are susceptible to all drugs while $b-s$ denotes the total population of bacteria that are resistant to at least one drug, and the red line delimits the safe set (bottom left), time evolution of the optimal normalised drug concentrations $u_j/{\bar{u}}$ (top right), time evolution of the cost functional *J* (bottom right) in the case with $m=3$ antibiotics, with initial conditions chosen as in scenario 2 in Table 2. The parameters are chosen as described in the main text. The final cost is $J_T={3.55\times 10^7}$.

Scenario 2 in Table 2 considers a drastically reduced amount of bacteria that are resistant to all antibiotics, and the presence of all possible combinations of resistance in variable amounts, with a prevalence of bacteria that are resistant to *B* and a comparatively small number of bacteria that are resistant to *C*: in this case, as shown in Figure 12, antibiotic *A* is still the preferred choice, but the administration duration reduces to 15.97 hours.

**Fig. 13:**
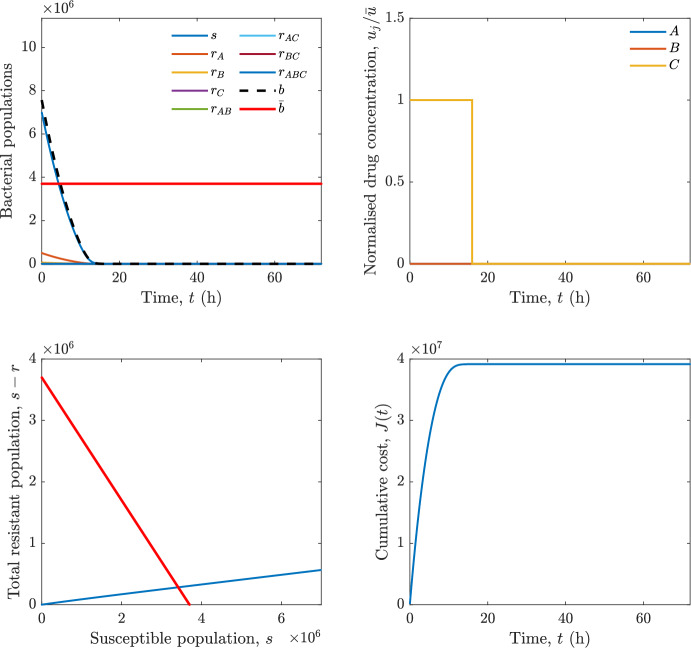
(OCP) Time evolution of the various bacterial populations, with the value of ${\bar{b}}$ marked by the red horizontal line (top left), system trajectory in the plane $b-s$ vs. *s*, where *s* are bacteria that are susceptible to all drugs while $b-s$ denotes the total population of bacteria that are resistant to at least one drug, and the red line delimits the safe set (bottom left), time evolution of the optimal normalised drug concentrations $u_j/{\bar{u}}$ (top right), time evolution of the cost functional *J* (bottom right) in the case with $m=3$ antibiotics, with initial conditions chosen as in scenario 3 in Table 2. The parameters are chosen as described in the main text. The final cost is $J_T={3.91\times 10^7}$.

Further increasing the number of bacteria that are resistant to *C* and, in particular, to *A* (while reducing the number of resistant to *B* only), as in scenario 3 in Table 2, leads to the administration of antibiotic *C* for 15.98 hours (see Figure 13): although less effective than *A*, it is preferred since the number of resistant bacteria to *A* has crossed the threshold beyond which the higher killing rate of antibiotic *A* cannot compensate for its inability to kill a significant fraction of the bacterial population.

**Fig. 14:**
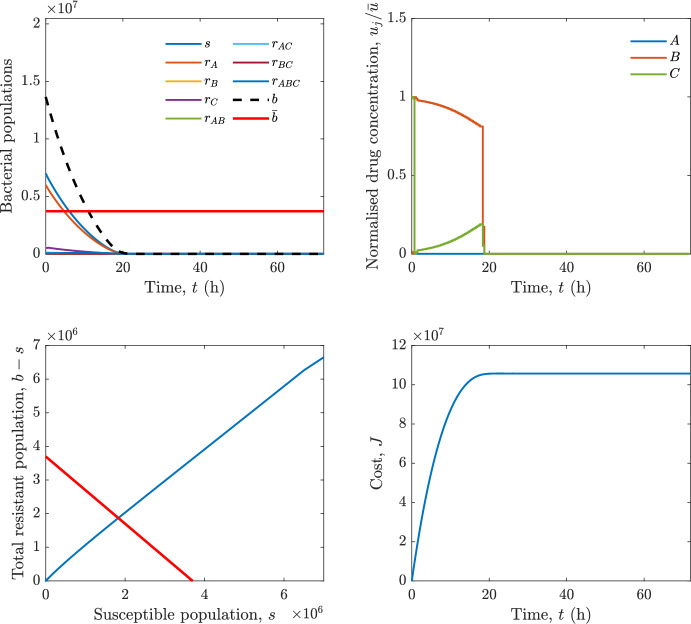
(OCP) Time evolution of the various bacterial populations, with the value of ${\bar{b}}$ marked by the red horizontal line (top left), system trajectory in the plane $b-s$ vs. *s*, where *s* are bacteria that are susceptible to all drugs while $b-s$ denotes the total population of bacteria that are resistant to at least one drug, and the red line delimits the safe set (bottom left), time evolution of the optimal normalised drug concentrations $u_j/{\bar{u}}$ (top right), time evolution of the cost functional *J* (bottom right) in the case with $m=3$ antibiotics, with initial conditions chosen as in scenario 4 in Table 2. The parameters are chosen as described in the main text. The final cost is $J_T={1.05\times 10^8}$.

In scenario 4 in Table 2, there are no bacteria that are resistant to *B* only, while the number of bacteria that are resistant to *A* and to *C* is further increased: as shown in Figure 14, this leads to the administration of antibiotic *C* for less than 1 hour, followed by a combination with antibiotic *B* (the least effective in terms of killing rate, but preferred because most bacteria are susceptible to it) for additional 18 hours. Also in this case, we can observe singular controls.

### Model Predictive Control Solution

Finding a solution to the treatment design problem through a model predictive control scheme (27) yields a sub-optimal solution with respect to solving the optimal control problem. However, computationally and practically, it introduces two key advantages: the reduced horizon makes each sub-problem numerically well-conditioned and fast to solve, and the periodic re-optimisation from the true state provides closed-loop feedback that inherently mitigates disturbances and unmodelled dynamics.

**Fig. 15:**
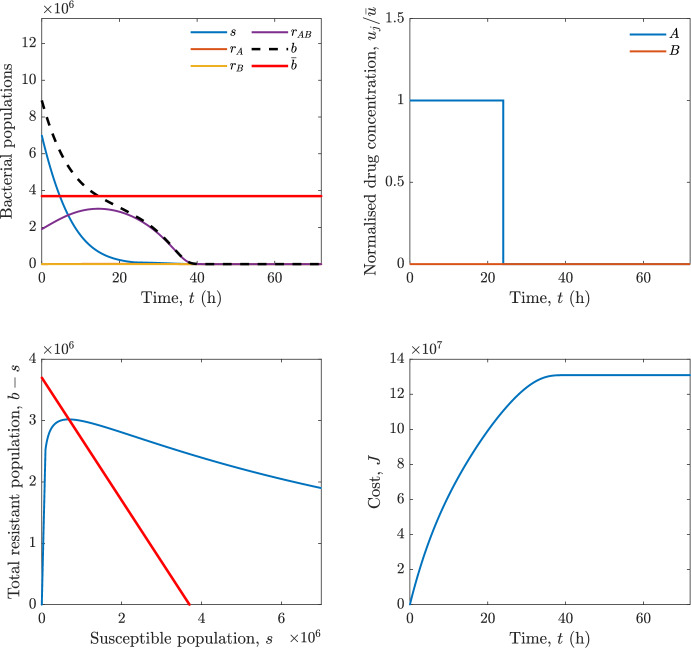
(MPC) Time evolution of the various bacterial populations, with the value of ${\bar{b}}$ marked by the red horizontal line (top left), system trajectory in the plane $b-s$ vs. *s*, where *s* are bacteria that are susceptible to all drugs while $b-s$ denotes the total population of bacteria that are resistant to at least one drug, and the red line delimits the safe set (bottom left), time evolution of the optimal normalised drug concentrations $u_j/{\bar{u}}$ (top right), time evolution of the cost functional *J* (bottom right) in the case with $m=2$ antibiotics, with initial conditions chosen as in scenario 1 in Table 2. The parameters are chosen as described in the main text. The final cost is $J_T={1.31\times 10^8}$.

**Fig. 16:**
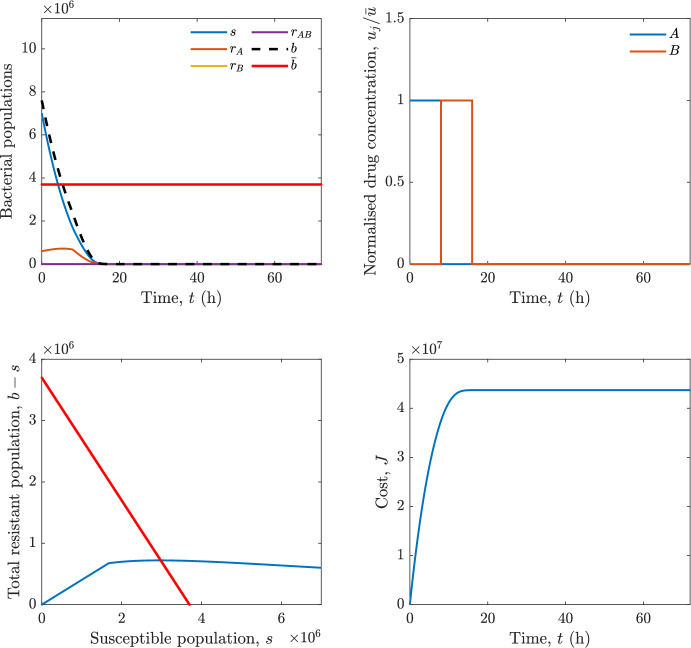
(MPC) Time evolution of the various bacterial populations, with the value of ${\bar{b}}$ marked by the red horizontal line (top left), system trajectory in the plane $b-s$ vs. *s*, where *s* are bacteria that are susceptible to all drugs while $b-s$ denotes the total population of bacteria that are resistant to at least one drug, and the red line delimits the safe set (bottom left), time evolution of the optimal normalised drug concentrations $u_j/{\bar{u}}$ (top right), time evolution of the cost functional *J* (bottom right) in the case with $m=2$ antibiotics, with initial conditions chosen as in scenario 2 in Table 2. The parameters are chosen as described in the main text. The final cost is $J_T={4.37\times 10^7}$.

**Fig. 17:**
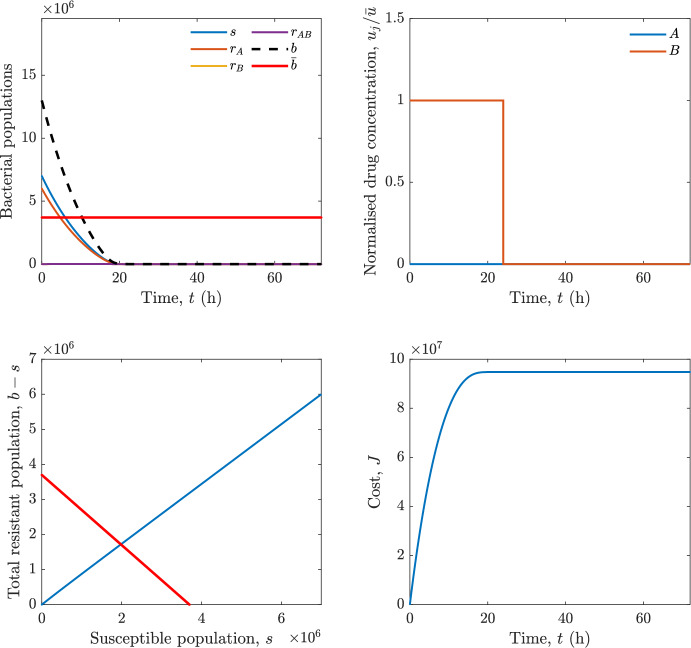
(MPC) Time evolution of the various bacterial populations, with the value of ${\bar{b}}$ marked by the red horizontal line (top left), system trajectory in the plane $b-s$ vs. *s*, where *s* are bacteria that are susceptible to all drugs while $b-s$ denotes the total population of bacteria that are resistant to at least one drug, and the red line delimits the safe set (bottom left), time evolution of the optimal normalised drug concentrations $u_j/{\bar{u}}$ (top right), time evolution of the cost functional *J* (bottom right) in the case with $m=2$ antibiotics, with initial conditions chosen as in scenario 3 in Table 2. The parameters are chosen as described in the main text. The final cost is $J_T={9.48\times 10^7}.$

**Fig. 18:**
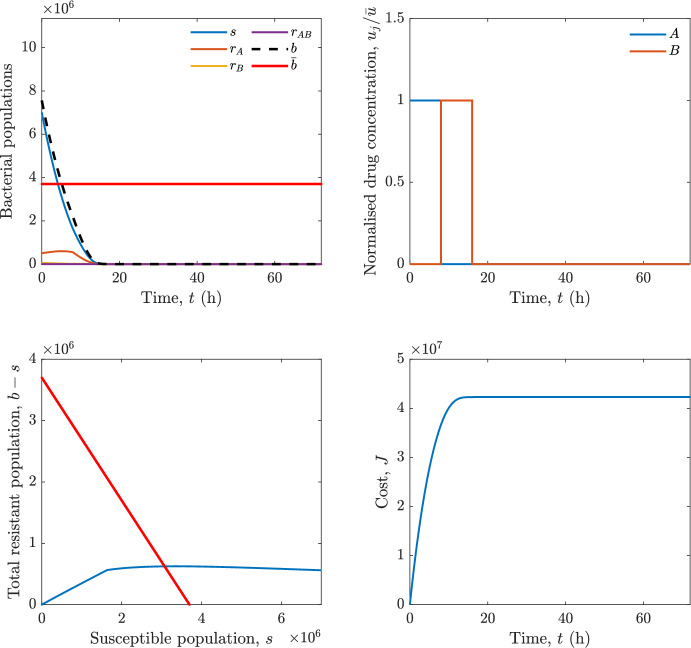
(MPC) Time evolution of the various bacterial populations, with the value of ${\bar{b}}$ marked by the red horizontal line (top left), system trajectory in the plane $b-s$ vs. *s*, where *s* are bacteria that are susceptible to all drugs while $b-s$ denotes the total population of bacteria that are resistant to at least one drug, and the red line delimits the safe set (bottom left), time evolution of the optimal normalised drug concentrations $u_j/{\bar{u}}$ (top right), time evolution of the cost functional *J* (bottom right) in the case with $m=2$ antibiotics, with initial conditions chosen as in scenario 4 in Table 2. The parameters are chosen as described in the main text. The final cost is $J_T={4.23\times 10^7}.$

**Fig. 19:**
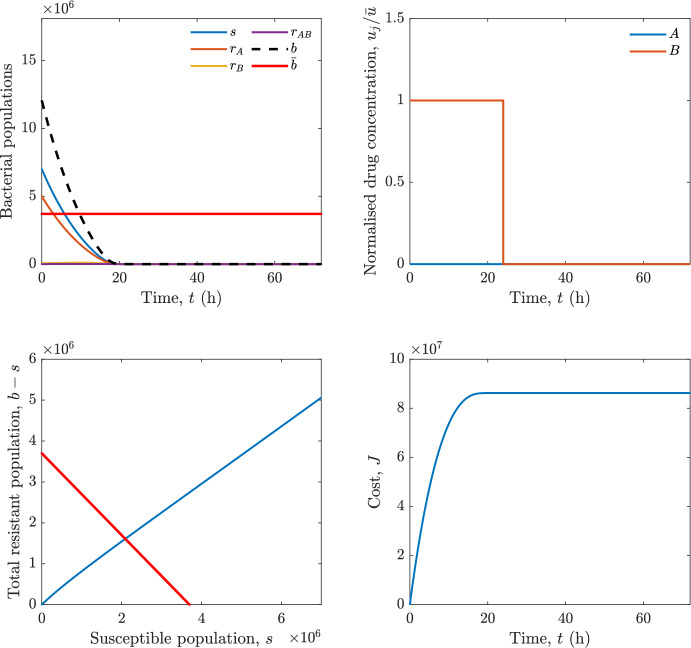
(MPC) Time evolution of the various bacterial populations, with the value of ${\bar{b}}$ marked by the red horizontal line (top left), system trajectory in the plane $b-s$ vs. *s*, where *s* are bacteria that are susceptible to all drugs while $b-s$ denotes the total population of bacteria that are resistant to at least one drug, and the red line delimits the safe set (bottom left), time evolution of the optimal normalised drug concentrations $u_j/{\bar{u}}$ (top right), time evolution of the cost functional *J* (bottom right) in the case with $m=2$ antibiotics, with initial conditions chosen as in scenario 5 in Table 2. The parameters are chosen as described in the main text. The final cost is $J_T={8.62\times 10^7}$.

**Fig. 20:**
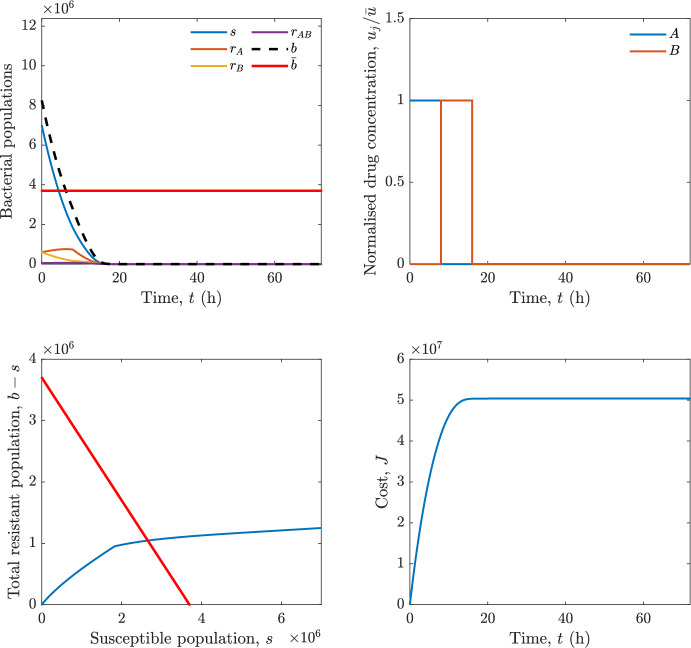
(MPC) Time evolution of the various bacterial populations, with the value of ${\bar{b}}$ marked by the red horizontal line (top left), system trajectory in the plane $b-s$ vs. *s*, where *s* are bacteria that are susceptible to all drugs while $b-s$ denotes the total population of bacteria that are resistant to at least one drug, and the red line delimits the safe set (bottom left), time evolution of the optimal normalised drug concentrations $u_j/{\bar{u}}$ (top right), time evolution of the cost functional *J* (bottom right) in the case with $m=2$ antibiotics, with initial conditions chosen as in scenario 6 in Table 2. The parameters are chosen as described in the main text. The final cost is $J_T={5.0385\times 10^7}$.

**Fig. 21:**
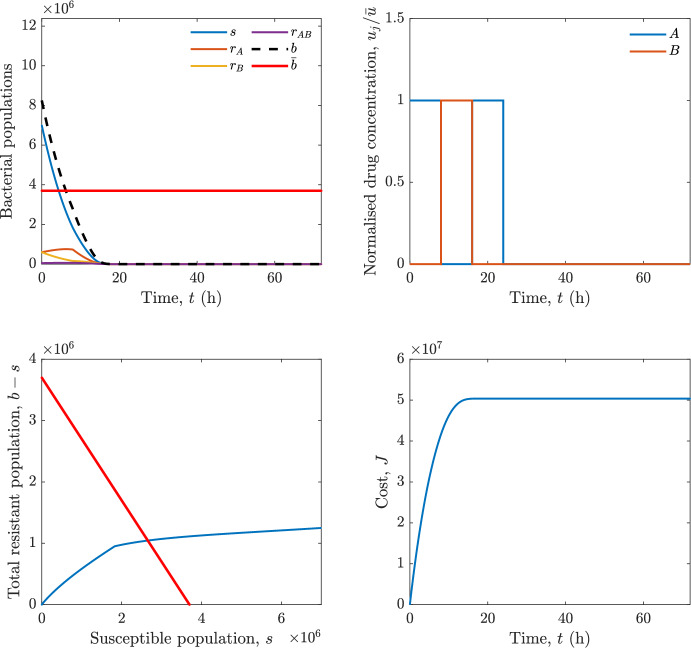
(MPC) Time evolution of the various bacterial populations, with the value of ${\bar{b}}$ marked by the red horizontal line (top left), system trajectory in the plane $b-s$ vs. *s*, where *s* are bacteria that are susceptible to all drugs while $b-s$ denotes the total population of bacteria that are resistant to at least one drug, and the red line delimits the safe set (bottom left), time evolution of the optimal normalised drug concentrations $u_j/{\bar{u}}$ (top right), time evolution of the cost functional *J* (bottom right) in the case with $m=2$ antibiotics, with initial conditions chosen as in scenario 6 in Table 2. The parameters are chosen as described in the main text, with the exception of $\lambda _i=0.001,\,i=1,2$. The final cost is $J_T={5.0381\times 10^7}$.

**Fig. 22:**
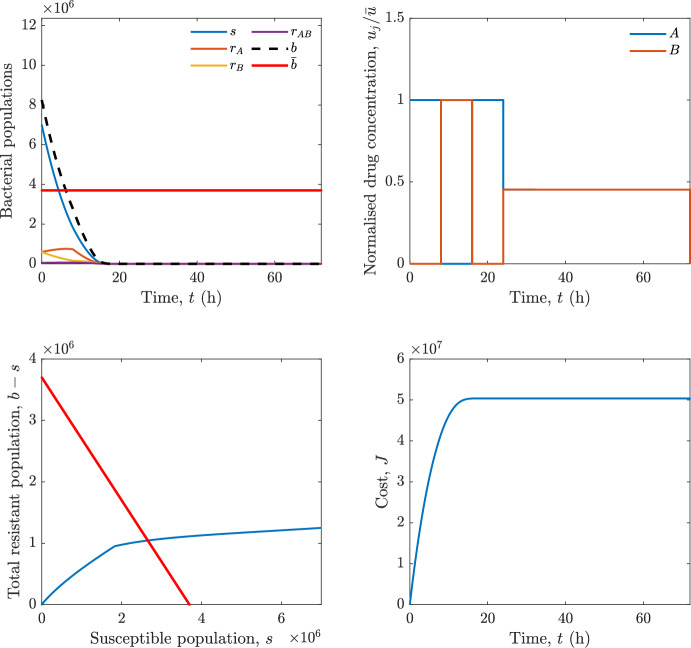
(MPC) Time evolution of the various bacterial populations, with the value of ${\bar{b}}$ marked by the red horizontal line (top left), system trajectory in the plane $b-s$ vs. *s*, where *s* are bacteria that are susceptible to all drugs while $b-s$ denotes the total population of bacteria that are resistant to at least one drug, and the red line delimits the safe set (bottom left), time evolution of the optimal normalised drug concentrations $u_j/{\bar{u}}$ (top right), time evolution of the cost functional *J* (bottom right) in the case with $m=2$ antibiotics, with initial conditions chosen as in scenario 6 in Table 2. The parameters are chosen as described in the main text, with the exception of $\lambda _i=0,\,i=1,2$. The final cost is $J_T={5.0381\times 10^7}$.

**Fig. 23:**
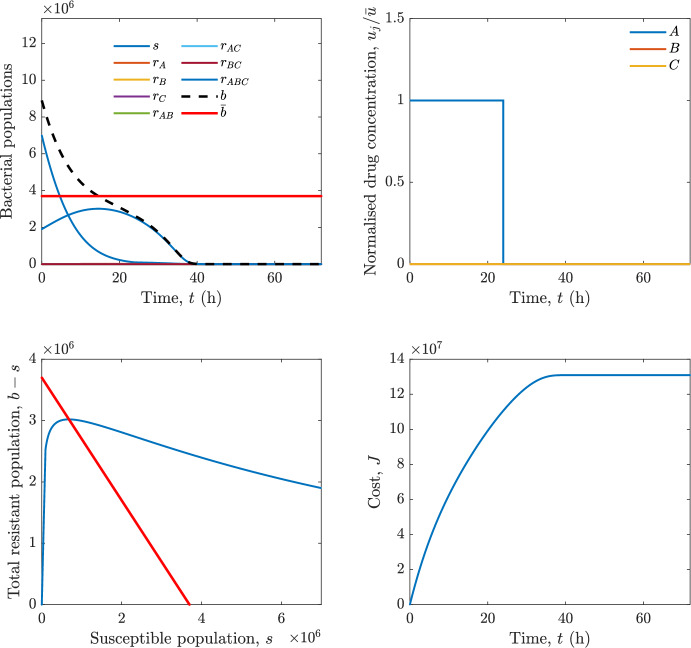
(MPC) Time evolution of the various bacterial populations, with the value of ${\bar{b}}$ marked by the red horizontal line (top left), system trajectory in the plane $b-s$ vs. *s*, where *s* are bacteria that are susceptible to all drugs while $b-s$ denotes the total population of bacteria that are resistant to at least one drug, and the red line delimits the safe set (bottom left), time evolution of the optimal normalised drug concentrations $u_j/{\bar{u}}$ (top right), time evolution of the cost functional *J* (bottom right) in the case with $m=3$ antibiotics, with initial conditions chosen as in scenario 1 in Table 2. The parameters are chosen as described in the main text. The final cost is $J_T={1.31\times 10^8}$.

**Fig. 24:**
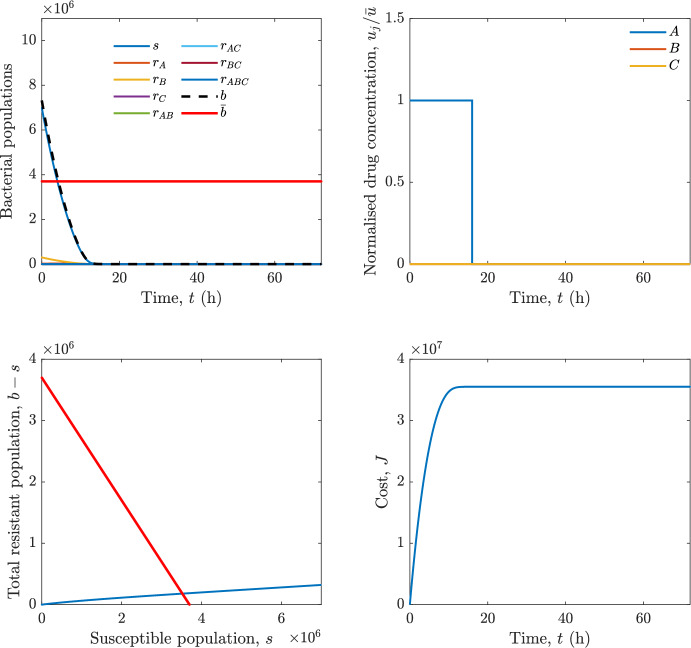
(MPC) Time evolution of the various bacterial populations, with the value of ${\bar{b}}$ marked by the red horizontal line (top left), system trajectory in the plane $b-s$ vs. *s*, where *s* are bacteria that are susceptible to all drugs while $b-s$ denotes the total population of bacteria that are resistant to at least one drug, and the red line delimits the safe set (bottom left), time evolution of the optimal normalised drug concentrations $u_j/{\bar{u}}$ (top right), time evolution of the cost functional *J* (bottom right) in the case with $m=3$ antibiotics, with initial conditions chosen as in scenario 2 in Table 2. The parameters are chosen as described in the main text. The final cost is $J_T={3.55\times 10^7}$.

**Fig. 25:**
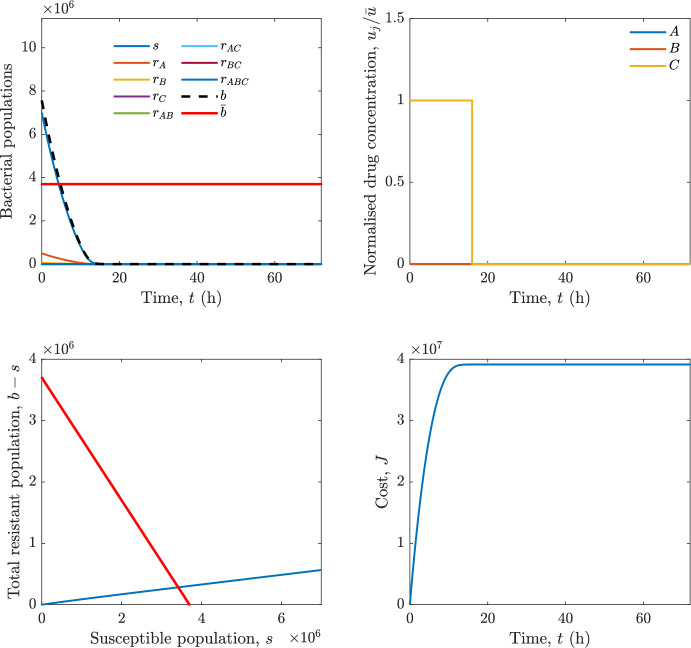
(MPC) Time evolution of the various bacterial populations, with the value of ${\bar{b}}$ marked by the red horizontal line (top left), system trajectory in the plane $b-s$ vs. *s*, where *s* are bacteria that are susceptible to all drugs while $b-s$ denotes the total population of bacteria that are resistant to at least one drug, and the red line delimits the safe set (bottom left), time evolution of the optimal normalised drug concentrations $u_j/{\bar{u}}$ (top right), time evolution of the cost functional *J* (bottom right) in the case with $m=3$ antibiotics, with initial conditions chosen as in scenario 3 in Table 2. The parameters are chosen as described in the main text. The final cost is $J_T={3.91\times 10^7}$.

**Fig. 26:**
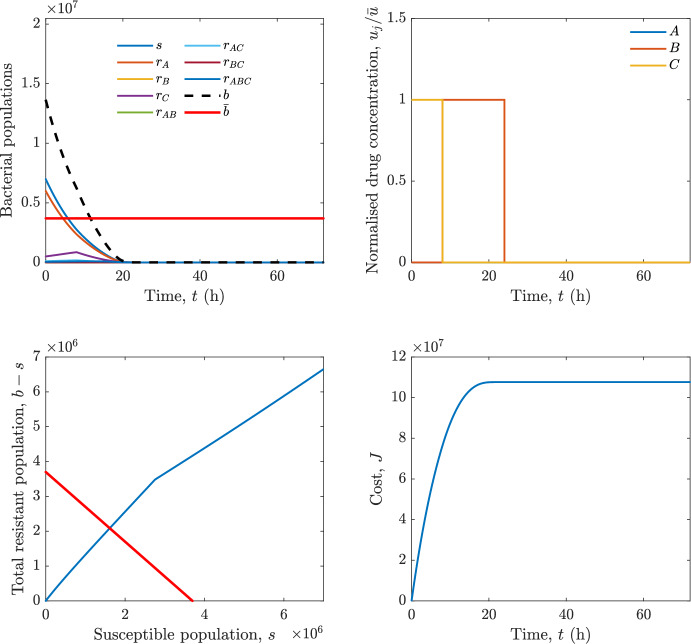
(MPC) Time evolution of the various bacterial populations, with the value of ${\bar{b}}$ marked by the red horizontal line (top left), system trajectory in the plane $b-s$ vs. *s*, where *s* are bacteria that are susceptible to all drugs while $b-s$ denotes the total population of bacteria that are resistant to at least one drug, and the red line delimits the safe set (bottom left), time evolution of the optimal normalised drug concentrations $u_j/{\bar{u}}$ (top right), time evolution of the cost functional *J* (bottom right) in the case with $m=3$ antibiotics, with initial conditions chosen as in scenario 4 in Table 2. The parameters are chosen as described in the main text. The final cost is $J_T={1.08\times 10^8}$.

For our numerical simulation campaign, again we consider two different case studies, one with $m=2$ antibiotics and the other with $m=3$ antibiotics, hereafter denoted as *A*, *B*, and (when relevant) *C*. The values of the model parameters are chosen as reported in Table 1, following realistic case studies in the biological literature [16]. The value of ${\bar{b}}$ defined in (3), computed according to the parameter values in Table 1, is $\bar{b}= 3.6997 \times 10^{6}$. We also set $\bar{u}=1.5\tfrac{\alpha }{\min _{j\in \{A,B,C\}}{\mu _P}} \approx 2.1889$ as the maximum tolerated drug concentration.

For the Euler discretisation, we choose $\tau _d=\tfrac{1}{60}$h, whereas, in the model predictive control scheme (27), we set the sampling time equal to $\tau ={8}$h and the prediction length equal to $N_{\text {h}}=60$ steps, so that the length of the prediction horizon is $N_{\text {h}} \tau _d = {1}$h. The weights in the cost functional are chosen as $\xi _1=\xi _2=1$ and $\lambda _i=0.1$, $i=1,2,3$. We consider a simulation window of 3 days, so that the whole therapy duration is equal to $T={72}$h. The considered initial conditions, corresponding to different scenarios, are reported in Table 2. The evolution of the state *x* and of the input *u* generated by the model predictive approach is obtained with MATLAB, using Yalmip [43] and the solver ipopt [8, 65], as well as using CasAdi [5] through its MATLAB interface (the obtained solutions are identical). We denote with $J_T$ the value of the cost functional corresponding to the optimal solution to (27).

In the case of $m=2$ antibiotics, we begin by considering scenario 1 in Table 2, in which only $7\times 10^{6}$ susceptible bacteria and $1.9\times 10^{6}$ bacteria that are resistant to both antibiotics are initially present, in Figure 15: as expected, antibiotic *A*, which has the highest killing rate, is chosen and is administered for 24 hours, up to short after the total number *b* of bacteria decreases below the threshold ${\bar{b}}$. Also in this case, since the aim is not only to steer the trajectories into the safe set, but also to minimise the final value of the state as well as the integral of the state, in all considered scenarios antibiotics are administered also after the trajectory has reached the safe set. Comparing the obtained solution with the one provided in the previous section for the related optimal control problem, the final cost is higher with MPC, although the drug is administered for a shorter period, because the optimal control formulation achieves a stronger reduction of the bacterial population.

An initial condition with a reduced amount of bacteria that are resistant to both antibiotics ($1\times 10^{3}$) and the additional significant presence of $6\times 10^{5}$ bacteria that are resistant to antibiotic *A*, in the absence of bacteria that are resistant to antibiotic *B* (scenario 2 in Table 2), leads to the administration of antibiotic *A*, for 8 hours, followed by antibiotic *B*, for additional 8 hours (Figure 16). This solution provides a different scheduling from the one obtained by solving the optimal control problem. Although sub-optimal, this protocol is clinically practical, since the antibiotic concentrations are re-assessed every 8 hours.

Further increasing the initial number of bacteria that are resistant to antibiotic *A* to $6\times 10^{6}$, *ceteris paribus* (scenario 3 in Table 2), leads to the administration of antibiotic *B* only, for 24 hours (Figure 17).

However, reducing the initial amount of bacteria that are resistant to *A* to $5\times 10^{5}$ and introducing $6\times 10^{4}$ bacteria that are resistant to *B* (scenario 4 in Table 2) leads again to the administration of antibiotic *A*, for 8 hours, followed by antibiotic *B*, for 8 hours (Figure 18). Also in this case, the singular controls are approximated by the MPC scheme with piecewise-constant functions, since the drug concentrations are kept constant for 8 hours.

Increasing the initial amount of bacteria that are resistant to *A* to $5\times 10^{6}$, while keeping $6\times 10^{4}$ bacteria that are resistant to *B* (scenario 5 in Table 2), leads again to the administration of antibiotic *B* for 24 hours (Figure 19).

Finally, for different choices of the weights in the cost functional, we consider scenario 6 in Table 2, corresponding to the coexistence, with $7\times 10^{6}$ bacteria that are susceptible, of bacteria that are resistant to *A* and resistant to *B* in the same number ($6\times 10^{5}$), along with $5\times 10^{4}$ bacteria that are resistant to both. Keeping $\lambda _i=0.1$, $i=1,2$ yields the administration of antibiotic *A* for 8 hours, followed by antibiotic *B* for 8 hours (Figure 20). The singular controls obtained by solving the optimal control problem are once again approximated, in the MPC solution, by piecewise-constant functions, reflecting the assumption that drug concentrations remain constant over periods of 8 hours. Decreasing the weight on the control to $\lambda _i=0.001$, $i=1,2$ leads to the administration of antibiotic *A* for 8 hours, followed by antibiotic *B* for 8 hours and then by antibiotic *A* again for additional 8 hours (Figure 21); if, furthermore, the control is not considered in the cost functional, an additional administration of antibiotic *B* at half of the maximum tolerated concentration is provided up to the end of the horizon (Figure 22).

The infection is cleared in all the considered case studies, within 40 hours (although drug administration stops after 24 hours in the MPC case and after 28 hours in the OCP case) in scenario 1, due to the significant amount of bacteria that are resistant to both antibiotics and hence can only be killed by the immune system cells, and within 20 hours in all other cases. The examples show that the optimal choice of the therapy, either according to the optimal control problem in (22) or to the MPC optimisation in (27), strongly depends not only on the chosen weights in the cost functional, but also on the initial conditions: an optimal treatment must account for the presence of resistant bacteria and for their number, and choose accordingly from the available antibiotics, possibly alternating among them.

In the case of $m=3$ antibiotics, when only $7\times 10^{6}$ susceptible bacteria and $1.9\times 10^{6}$ bacteria that are resistant to all antibiotics are initially present (scenario 1 in Table 2), antibiotic *A* is administered for 24 hours, which is again expected since it is the most effective (i.e., it has the highest killing rate) of the three available antibiotics; see Figure 23. Also in this case, the MPC solution replaces the singular controls that solve the optimal control problem with piecewise-constant functions.

Scenario 2 in Table 2 considers a drastically reduced amount of bacteria that are resistant to all antibiotics, and the presence of all possible combinations of resistance in variable amounts, with a prevalence of bacteria that are resistant to *B* and a comparatively small number of bacteria that are resistant to *C*: in this case, as shown in Figure 24, antibiotic *A* is still the preferred choice, but the administration duration reduces to 16 hours.

Further increasing the number of bacteria that are resistant to *C* and, in particular, to *A* (while reducing the number of resistant to *B* only), as in scenario 3 in Table 2, leads to the administration of antibiotic *C* for 16 hours (see Figure 25): although less effective than *A*, it is preferred since the number of resistant bacteria to *A* has crossed the threshold beyond which the higher killing rate of antibiotic *A* cannot compensate for its inability to kill a significant fraction of the bacterial population.

In scenario 4 in Table 2, there are no bacteria that are resistant to *B* only, while the number of bacteria that are resistant to *A* and to *C* is further increased: as shown in Figure 26, this leads to the administration of antibiotic *C* for 8 hours, followed by antibiotic *B* (the least effective in terms of killing rate, but preferred because most bacteria are susceptible to it) for additional 16 hours. As in the optimal control solution, only drugs *B* and *C* are administered.

Also with $m=3$ drugs, the infection is cleared in all the considered case studies, within 40 hours (although drug administration stops after 24 hours) in scenario 1, due to the significant amount of bacteria that are resistant to all antibiotics and hence can only be killed by the immune system cells, and within 20 hours in all other cases. In this case too, the optimal choice of the therapy strongly depends on the initial conditions, and therapies that alternate among available antibiotics can emerge as the optimal ones.

## Concluding Discussion

We have proposed a flexible framework for the optimal planning of antibiotic therapies in the presence of bacterial infections where antimicrobial resistance arises and spreads due to treatment-induced mutations and horizontal transfer, through a model that also accounts for the effect of the immune system. After thoroughly analysing the model properties, we have proven the existence of a solution to the formulated optimal control problem and we have analysed it, providing necessary conditions for optimality through the Pontryagin minimum principle, as well as singular controls. We have also proposed an alternative approach to design therapies through a finite-horizon model predictive control scheme. For both the optimal control problem and the MPC approach, we have demonstrated our theoretical results through a campaign of numerical simulations. Our numerical results have emphasised the importance, in determining the optimal therapeutic strategy, not only of the weights of the cost functional, but also of the initial conditions of the system; also, optimal therapies that alternate among (some of) the available antibiotics have naturally emerged.

This is consistent with existing observations. By adjusting treatment strategies, such as timing, dosage, and duration of therapy, the bacterial population size can be reduced effectively while limiting the selection pressure that drives the emergence of resistant strains. In particular, combination therapies, which involve the use of multiple drugs with different mechanisms of action, have been shown to significantly decrease the likelihood of resistance onset compared to monotherapies [16, 35], provided that they are chosen carefully to avoid combining reciprocal suppressive drugs [36]. Additionally, cycling therapies, where different drugs (or drug combinations) are rotated over time, can reduce selective pressure on bacteria to develop resistance to a single agent [2, 4, 30, 60, 61]; similar optimal switching strategies have also been proposed in the context of antiviral therapies [28, 29] and cancer treatment [18, 25]. This type of therapeutic strategies helps limit the persistence of resistant bacterial populations by alternating the mechanisms of action, thereby improving long-term treatment outcomes and preserving the efficacy of existing drugs.

Future work aims at applying the developed framework to design optimal therapeutic strategies in experimental settings, after fitting the model parameters related to the evolution of bacterial populations to time series acquired through experiments *in vitro*.

## Data Availability

The manuscript has no associated data. All information needed to ensure reproducibility of the numerical results is included in the manuscript.

## A Derivation of Singular Controls

For a more concise notation, we rename the following quantities

$$\begin{aligned} f_0(x) {:}{=} \psi (b)\,x, \qquad f_A(x) {:}{=} F_A(x)\,x, \qquad f_B(x) {:}{=} F_B(x)\,x, \end{aligned}$$

and the Jacobians of the control vector fields are:

$$\begin{aligned} J_A {:}{=} \frac{\partial f_A}{\partial x} = F_A(x) + (\nabla _x F_A(x))\,x, \qquad J_B {:}{=} \frac{\partial f_B}{\partial x} = F_B(x) + (\nabla _x F_B(x))\,x. \end{aligned}$$

Then, the switching functions become:

$$\begin{aligned} \sigma _A = \xi _1\lambda _1 + p^\top f_A(x), \qquad \sigma _B = \xi _1\lambda _2 + p^\top f_B(x). \end{aligned}$$

### A.1 Derivation of First-Order Conditions

Differentiating $\sigma _K = \xi _1\lambda _K + p^\top f_K$ along the optimal trajectory yields

$$\begin{aligned} {\dot{\sigma }}_K = \dot{p}^\top f_K + p^\top J_K \dot{x}. \end{aligned}$$

It is

$$\begin{aligned} \dot{p}^\top f_K = -\xi _1{\textbf {1}}^\top f_K - \psi '(b)(p^\top x)({\textbf {1}}^\top f_K) - \psi (b)(p^\top f_K) = - u_A\,p^\top J_A f_K - u_B\,p^\top J_B f_K \end{aligned}$$

and

$$\begin{aligned} p^\top J_K \dot{x} = \psi (b)\,p^\top J_K x + u_A\,p^\top J_K f_A + u_B\,p^\top J_K f_B. \end{aligned}$$

Summing for $K = A$, the coefficient of $u_A$ is $-p^\top J_A f_A + p^\top J_A f_A = 0$. Thus, the diagonal terms cancel out exactly. The coefficient of $u_B$ is $-p^\top J_B f_A + p^\top J_A f_B = q$. Similarly, for $K = B$, the coefficient of $u_B$ cancels out, while the coefficient of $u_A$ is $-q$.

### A.2 Derivation of Second-Order Conditions

We introduce two auxiliary scalars. Using $p^\top f_A = -\xi _1\lambda _1$ and $p^\top f_B = -\xi _1\lambda _2$ from (24):

$$\begin{aligned} \eta _A&{:}{=} p^\top J_A x - \xi _1\lambda _1 = p^\top J_A x + p^\top f_A, \\ \eta _B&{:}{=} p^\top J_B x - \xi _1\lambda _2 = p^\top J_B x + p^\top f_B. \end{aligned}$$

The time derivative of $b = {\textbf {1}}^\top x$ is

28 $$\begin{aligned} \dot{b} = \psi (b)\,b + u_A\,{\textbf {1}}^\top f_A + u_B\,{\textbf {1}}^\top f_B. \end{aligned}$$

The time derivative of $p^\top x$ is

29 $$\begin{aligned} \frac{d}{dt}(p^\top x) = -\xi _1 b - \psi '(b)(p^\top x)\,b - u_A\,\eta _A - u_B\,\eta _B. \end{aligned}$$

### A.3 Differentiation of $C_A$

We write $C_A = D_1 + D_2 + D_3 + D_4$, with

$$\begin{aligned}&D_1 = -\xi _1{\textbf {1}}^\top f_A, \quad D_2 = -\psi '(b)(p^\top x)({\textbf {1}}^\top f_A), \quad D_3 = -\psi (b)(p^\top f_A), \quad \\&D_4 = \psi (b)\,p^\top J_A x. \end{aligned}$$

Since $D_1 = -\xi _1{\textbf {1}}^\top f_A(x)$, we have

$$\begin{aligned} \dot{D}_1 = -\xi _1\psi (b)\,{\textbf {1}}^\top J_A x - \xi _1 u_A\,{\textbf {1}}^\top J_A f_A - \xi _1 u_B\,{\textbf {1}}^\top J_A f_B. \end{aligned}$$

Differentiating the product $D_2 = -\psi '(b)(p^\top x)({\textbf {1}}^\top f_A)$ and substituting (28), (29), and $\frac{d({\textbf {1}}^\top f_A)}{dt} = {\textbf {1}}^\top J_A\dot{x}$, we get

$$\begin{aligned} \dot{D}_2&= -\psi ''(b)\psi (b) b\,(p^\top x)({\textbf {1}}^\top f_A) + \psi '(b)\bigl [\xi _1 b + \psi '(b)(p^\top x)b\bigr ]({\textbf {1}}^\top f_A) \\&- \psi '(b)(p^\top x)\psi (b)\,{\textbf {1}}^\top J_A x \nonumber \\&\quad + u_A\Bigl [ -\psi ''(b)({\textbf {1}}^\top f_A)(p^\top x)({\textbf {1}}^\top f_A) + \psi '(b)\eta _A({\textbf {1}}^\top f_A) - \psi '(b)(p^\top x)\,{\textbf {1}}^\top J_A f_A \Bigr ] \nonumber \\&\quad + u_B\Bigl [ -\psi ''(b)({\textbf {1}}^\top f_B)(p^\top x)({\textbf {1}}^\top f_A) + \psi '(b)\eta _B({\textbf {1}}^\top f_A) - \psi '(b)(p^\top x)\,{\textbf {1}}^\top J_A f_B \Bigr ]. \end{aligned}$$

Since $p^\top f_A = -\xi _1\lambda _1$ on the singular arc, $\frac{d}{dt}(p^\top f_A) = {\dot{\sigma }}_A = C_A = 0$. Therefore,

$$\begin{aligned} \dot{D}_3 = -\psi '(b)\dot{b}\,(p^\top f_A) = \xi _1\lambda _1\psi '(b)\bigl [\psi b + u_A({\textbf {1}}^\top f_A) + u_B({\textbf {1}}^\top f_B)\bigr ] \end{aligned}$$

and

$$\begin{aligned} \dot{D}_4 = \psi '(b)\dot{b}\,(p^\top J_A x) + \psi (b)\,\frac{d}{dt}(p^\top J_A x). \end{aligned}$$

For the second factor,

$$\begin{aligned} \frac{d}{dt}(p^\top J_A x) = \dot{p}^\top J_A x + p^\top (\partial _x J_A[\dot{x}])\,x + p^\top J_A\dot{x}. \end{aligned}$$

Using the costate equation (23), we obtain

$$\begin{aligned} \dot{p}^\top J_A x&= -\xi _1{\textbf {1}}^\top J_A x - \psi '(b)(p^\top x){\textbf {1}}^\top J_A x - \psi (b)\,p^\top J_A x \\&\quad - u_A\,p^\top J_A^\top J_A x - u_B\,p^\top J_B^\top J_A x, \end{aligned}$$

and using $\dot{x}$ from (12) yields

$$\begin{aligned} p^\top (\partial _x J_A[\dot{x}])\,x&= \psi \,p^\top (\partial _x J_A[x])\,x + u_A\,p^\top (\partial _x J_A[f_A])\,x + u_B\,p^\top (\partial _x J_A[f_B])\,x, \\ p^\top J_A\dot{x}&= \psi \,p^\top J_A x + u_A\,p^\top J_A f_A + u_B\,p^\top J_A f_B. \end{aligned}$$

Collecting all terms in $\dot{D}_4$, we have

$$\begin{aligned} \dot{D}_4&= \psi '(b)\psi b\,(p^\top J_A x) - \xi _1\psi \,{\textbf {1}}^\top J_A x - \psi '(b)(p^\top x)\psi \,{\textbf {1}}^\top J_A x + \psi (b)\bigl [\psi \,p^\top (\partial _x J_A[x])\,x\bigr ] \nonumber \\&\quad + u_A\Bigl [ \psi '(b)({\textbf {1}}^\top f_A)(p^\top J_A x) - \psi (b)\,p^\top J_A^\top J_A x + \psi (b)\,p^\top (\partial _x J_A[f_A])\,x + \psi (b)\,p^\top J_A f_A \Bigr ] \nonumber \\&\quad + u_B\Bigl [ \psi '(b)({\textbf {1}}^\top f_B)(p^\top J_A x) - \psi (b)\,p^\top J_B^\top J_A x + \psi (b)\,p^\top (\partial _x J_A[f_B])\,x + \psi (b)\,p^\top J_A f_B \Bigr ]. \end{aligned}$$

Summing $\dot{D}_1 + \dot{D}_2 + \dot{D}_3 + \dot{D}_4$ and collecting drift and control terms, we obtain

$$\begin{aligned} \ddot{\sigma }_A = {\tilde{C}}_A + {\tilde{Q}}_{AA}\,u_A + {\tilde{Q}}_{AB}\,u_B = 0, \end{aligned}$$

where the drift ${\tilde{C}}_A$ collects all terms independent of $u_A, u_B$, while

$$\begin{aligned} {\tilde{Q}}_{AA}&= - \xi _1\,{\textbf {1}}^\top J_A f_A - \psi ''(b)({\textbf {1}}^\top f_A)^2(p^\top x) + \psi '(b)\eta _A({\textbf {1}}^\top f_A) \nonumber \\&\quad - \psi '(b)(p^\top x)\,{\textbf {1}}^\top J_A f_A + \xi _1\lambda _1\psi '(b)({\textbf {1}}^\top f_A) \nonumber \\&\quad + \psi '(b)({\textbf {1}}^\top f_A)(p^\top J_A x) + \psi (b)\bigl (-p^\top J_A^\top J_A x + p^\top (\partial _x J_A[f_A])\,x + p^\top J_A f_A\bigr ), \end{aligned}$$

and

$$\begin{aligned} {\tilde{Q}}_{AB}&= - \xi _1\,{\textbf {1}}^\top J_A f_B - \psi ''(b)({\textbf {1}}^\top f_B)({\textbf {1}}^\top f_A)(p^\top x) + \psi '(b)\eta _B({\textbf {1}}^\top f_A) \nonumber \\&\quad - \psi '(b)(p^\top x)\,{\textbf {1}}^\top J_A f_B + \xi _1\lambda _1\psi '(b)({\textbf {1}}^\top f_B) \nonumber \\&\quad + \psi '(b)({\textbf {1}}^\top f_B)(p^\top J_A x) + \psi (b)\bigl (-p^\top J_B^\top J_A x + p^\top (\partial _x J_A[f_B])\,x + p^\top J_A f_B\bigr ). \end{aligned}$$

The same computation with *A* and *B* exchanged yields

$$\begin{aligned} \ddot{\sigma }_B = {\tilde{C}}_B + {\tilde{Q}}_{BA}\,u_A + {\tilde{Q}}_{BB}\,u_B = 0, \end{aligned}$$

with

$$\begin{aligned} {\tilde{Q}}_{BA}&= - \xi _1\,{\textbf {1}}^\top J_B f_A - \psi ''(b)({\textbf {1}}^\top f_A)({\textbf {1}}^\top f_B)(p^\top x) + \psi '(b)\eta _A({\textbf {1}}^\top f_B) \nonumber \\&\quad - \psi '(b)(p^\top x)\,{\textbf {1}}^\top J_B f_A + \xi _1\lambda _2\psi '(b)({\textbf {1}}^\top f_A) \nonumber \\&\quad + \psi '(b)({\textbf {1}}^\top f_A)(p^\top J_B x) + \psi (b)\bigl (-p^\top J_A^\top J_B x + p^\top (\partial _x J_B[f_A])\,x + p^\top J_B f_A\bigr ), \end{aligned}$$

and

$$\begin{aligned} {\tilde{Q}}_{BB}&= - \xi _1\,{\textbf {1}}^\top J_B f_B - \psi ''(b)({\textbf {1}}^\top f_B)^2(p^\top x) + \psi '(b)\eta _B({\textbf {1}}^\top f_B) \nonumber \\&\quad - \psi '(b)(p^\top x)\,{\textbf {1}}^\top J_B f_B + \xi _1\lambda _2\psi '(b)({\textbf {1}}^\top f_B) \nonumber \\&\quad + \psi '(b)({\textbf {1}}^\top f_B)(p^\top J_B x) + \psi (b)\bigl (-p^\top J_B^\top J_B x + p^\top (\partial _x J_B[f_B])\,x + p^\top J_B f_B\bigr ). \end{aligned}$$

The derivation of mixed arcs follows from the general case discussed above, and is hence omitted.
